# The Effect of Dosing Regimens on the Antimalarial Efficacy of Dihydroartemisinin-Piperaquine: A Pooled Analysis of Individual Patient Data

**DOI:** 10.1371/journal.pmed.1001564

**Published:** 2013-12-03

**Authors:** The WorldWide Antimalarial Resistance Network (WWARN) DP Study Group

**Affiliations:** Liverpool School of Tropical Medicine, United Kingdom

## Abstract

**Background:**

Dihydroartemisinin-piperaquine (DP) is increasingly recommended for antimalarial treatment in many endemic countries; however, concerns have been raised over its potential under dosing in young children. We investigated the influence of different dosing schedules on DP's clinical efficacy.

**Methods and Findings:**

A systematic search of the literature was conducted to identify all studies published between 1960 and February 2013, in which patients were enrolled and treated with DP. Principal investigators were approached and invited to share individual patient data with the WorldWide Antimalarial Resistance Network (WWARN). Data were pooled using a standardised methodology. Univariable and multivariable risk factors for parasite recrudescence were identified using a Cox's regression model with shared frailty across the study sites. Twenty-four published and two unpublished studies (*n* = 7,072 patients) were included in the analysis. After correcting for reinfection by parasite genotyping, Kaplan–Meier survival estimates were 97.7% (95% CI 97.3%–98.1%) at day 42 and 97.2% (95% CI 96.7%–97.7%) at day 63. Overall 28.6% (979/3,429) of children aged 1 to 5 years received a total dose of piperaquine below 48 mg/kg (the lower limit recommended by WHO); this risk was 2.3–2.9-fold greater compared to that in the other age groups and was associated with reduced efficacy at day 63 (94.4% [95% CI 92.6%–96.2%], *p*<0.001). After adjusting for confounding factors, the mg/kg dose of piperaquine was found to be a significant predictor for recrudescence, the risk increasing by 13% (95% CI 5.0%–21%) for every 5 mg/kg decrease in dose; *p* = 0.002. In a multivariable model increasing the target minimum total dose of piperaquine in children aged 1 to 5 years old from 48 mg/kg to 59 mg/kg would halve the risk of treatment failure and cure at least 95% of patients; such an increment was not associated with gastrointestinal toxicity in the ten studies in which this could be assessed.

**Conclusions:**

DP demonstrates excellent efficacy in a wide range of transmission settings; however, treatment failure is associated with a lower dose of piperaquine, particularly in young children, suggesting potential for further dose optimisation.

*Please see later in the article for the Editors' Summary*

## Abstract 2

Ric Price and colleagues pool individual patient data from efficacy trials of dihydroartemisinin-piperaquine shared with WWARN (Worldwide Antimalarial Resistance Network) to examine the potential for underdosing in young children.

*Please see later in the article for the Editors' Summary*

## Editors' Summary

**Background:**

Half of the world's population is at risk of malaria, a mosquito-borne parasitic disease that kills a million people (mainly young children in sub-Saharan Africa) every year. During the second half of the 20th century, the main treatments for malaria were “monotherapies” such as chloroquine and sulfadoxine-pyrimethamine. Unfortunately, parasitic resistance to these drugs rapidly spread and, in the 1990s, there was an upsurge in the illness and death caused by *Plasmodium falciparum*, the parasite responsible for most malarial deaths. To combat this increase, the World Health Organization (WHO) now recommends artemisinin-based combination therapy (ACT) for first-line treatment of *P. falciparum* malaria wherever malaria is endemic (always present). In ACT, artemisinin derivatives (antimalarial drugs that rapidly reduce the parasitic load in the patient and that are rapidly cleared from the body) are used in combination with a slower acting, more slowly eliminated partner drug that prevents recrudescent infections (re-emergences of the original infection).

**Why Was This Study Done?:**

ACT reduces the chances of *P. falciparum* becoming resistant to either the artemisinin derivative or the partner drug, but sub-optimal dosing can result in incomplete elimination of the initial parasitic infection and/or recrudescence. Both these situations drive the selection of parasites with reduced drug susceptibility. Unfortunately, current dosing strategies are usually based on weight or age bands that were determined during early drug development. Inevitably, some patients at the margins of these bands receive inappropriate doses—either too high or too low. Moreover, the doses recommended for children are extrapolated from adult doses and may not be optimal because children are not merely small adults—their drug responses often differ from those of adults. Here, researchers from the WorldWide Antimalarial Resistance Network (WWARN) investigate the influence of different dosing schedules on the clinical efficacy of dihydroartemisinin-piperaquine (DP, an ACT first recommended by WHO for the treatment of uncomplicated *P. falciparum* malaria in 2010) by undertaking a pooled analysis of individual patient data collected during ACT clinical studies.

**What Did the Researchers Do and Find?:**

The researchers identified all the studies published between 1960 and February 2013 in which patients were treated with DP and obtained individual patient data for almost 70% of study participants (more than 7,000 patients living in Asia, Africa, and South America) from the principal investigators. These data were pooled and statistical models were used to identify risk factors for parasite recrudescence (parasite genotyping was used to correct for re-infection with a new parasite). Overall, DP treatment was successful (judged by parasitological clearance) in 97.7% and 97.2% of patients at day 42 and 63, respectively, after treatment. However, 28.6% of children aged 1–5 years received a total dose of piperaquine over the 3-day DP dosing schedule of below 48 mg/kg body weight (the lower limit of dosing recommended by WHO). Children aged 1–5 years had a 2.9-fold higher risk of receiving a dose of piperaquine below 48 mg/kg than children aged 5–12 years. Moreover, the piperaquine dose was a significant predictor of recrudescence. For every 5 mg/kg decrease in dose, the risk of recrudescence increased by 13%. Finally, the researchers estimated that increasing the target dose of piperaquine in children aged 1–5 years to 59 mg/kg would halve the risk of treatment failure and cure at least 95% of young children.

**What Do These Findings Mean?:**

These findings suggest that DP treatment of malaria has a good efficacy in a wide range of settings but indicate that young children are at a higher risk of treatment failure than the overall population and that treatment failure, particularly in young children, is associated with a lower dose of piperaquine. Although the accuracy of these findings may be limited by some aspects of this study (for example, drug doses could only be calculated from the actual tablets taken in a quarter of the patients; in the remainder, they were extrapolated from the relevant study protocol), they nevertheless suggest that further detailed dose optimization studies in young children are essential to prolong the useful therapeutic life of DP. Moreover, if global elimination of malaria is to be achieved, similar pooled analyses to assess the efficacy of other drug combinations should be undertaken to ensure that ACT remains therapeutically useful for as long as possible.

**Additional Information:**

Please access these websites via the online version of this summary at http://dx.doi.org/10.1371/journal.pmed.1001564.

- This study is further discussed in a *PLoS Medicine* Perspective by Paul Garner
- Information is available from the World Health Organization on malaria (in several languages); the 2012 World Malaria Report provides details of the current global malaria situation; the 2010 WHO Guidelines for the Treatment of Malaria are available
- The US Centers for Disease Control and Prevention provide information on malaria (in English and Spanish), including a selection of personal stories about malaria
- Information is available from the Roll Back Malaria Partnership on the global control of malaria including fact sheets about ACT
- MedlinePlus provides links to additional information on malaria (in English and Spanish)
- Information on the WorldWide Antimalarial Resistance Network is available

## Introduction

Malaria is one of the leading causes of morbidity and mortality in endemic countries. Children under the age of 5 years are particularly vulnerable to failing treatment and developing severe disease, usually attributed to their lower immunity and premunition compared to older patients [1]–[3]. Prompt administration of highly effective antimalarial treatment can help to ensure parasitological cure, decrease transmission, and reduce the risk of complications and death; this approach is a key component of current elimination efforts [4]. The current consensus amongst policy makers advocates for artemisinin combination therapy (ACT) to slow the emergence and spread of antimalarial drug resistance [5]. This policy has been adopted in over 80 malaria-endemic countries for patients with uncomplicated *falciparum* malaria [6]. The high potency of the artemisinin component results in a rapid initial reduction in parasite biomass, with the sustained activity of the more slowly eliminated partner drug preventing subsequent recrudescent infections. Highly effective antimalarial treatment requires the administration of an optimised treatment regimen tailored to the weight and age of the patient. Sub-optimal dosing of either component can result in incomplete elimination of the parasite biomass and subsequent recrudescence, both of which are important driving forces for the selection of parasites with reduced drug susceptibility [7],[8].

The combination of dihydroartemisinin-piperaquine (DP) has been assessed in clinical trials for almost a decade, and shown to be highly efficacious against both *Plasmodium falciparum* and *P. vivax* infections [3],[9],[10]. Since 2010, DP has been recommended by WHO for the treatment of uncomplicated *falciparum* malaria [5], providing a promising alternative to other currently available ACTs, based upon its high efficacy, excellent safety profile, once daily dosing scheme, and prolonged post-treatment prophylactic protection [11]–[13]. The target doses of both dihydroartemisinin (DHA) and piperaquine (PIP) are usually quoted as the total mg/kg dose taken during the 3-day regimen. As is often the case, these recommendations were developed empirically in the early stages of the drug development with additional evidence provided by subsequent sparse pharmacokinetic data [14],[15]. In clinical practice, dosing strategies are usually pragmatic and based upon set weight or age banding. The inevitable consequence is that patients at the margins of these bands receive either lower or higher weight adjusted dosages. Furthermore, paediatric doses are often extrapolated from adult doses, since detailed pharmacokinetic data in children are frequently unavailable [15]. Thus, as detailed studies of a newly introduced drug are conducted, re-assessment of the original dosing recommendations based on a much larger body of evidence are needed to ensure that the original target doses are optimal for the key target populations, most notably the young children who carry the highest burden of disease in high intensity malaria transmission areas.

The Worldwide Antimalarial Resistance Network (WWARN) brings together malaria researchers from across the world. This collaborative resource provides a unique opportunity to conduct a series of individual patient data meta-analyses in which the dosing strategies of the recommended ACTs can be reviewed, to define the spectrum of treatment doses actually administered, and the degree to which these dose variations impact on the therapeutic efficacy [16]. The size of these pooled analyses provides unprecedented power and novel insights into the determinants of antimalarial efficacy. The first of these studies is focussed on the combination DP to explore the relationship between weight adjusted drug dosage (mg/kg) and therapeutic efficacy.

## Methods

### Data Pooling

All published antimalarial clinical trials reported in PubMed were identified through a systematic search of the literature of publications between 1st January 1960 and 15th February 2013. A further search was then made for those studies enrolling patients and treating them with DP. Further information on these literature reviews are available on the WWARN website [16]. Specific details on the relevant DP studies are presented in Texts S1 and S2 [11]–[13],[17]–[37]. Since the literature review was conducted, a further three clinical studies enrolling 408 patients treated with DP have been published. All research groups who have contributed to the WWARN data repository were also asked whether they were aware of any unpublished or ongoing clinical trials involving DP. Individual study protocols were available for all trials, either from the publication or as a metafile submitted with the raw data. The principal investigators of relevant studies were invited to contribute individual patient data to WWARN for a collaborative meta-analysis. A study was deemed eligible for the meta-analysis if patients were recruited and treated with DP and evaluated prospectively for clinical efficacy against *P. falciparum* (either alone or mixed infections), for a minimum of 28 days. Studies were included only if information was available on the treatment dose administered, the age and weight of the patient, and if genotyping was performed to distinguish between new infections and recrudescent infections. Both randomized and non-randomized studies were included. Data were anonymised, uploaded to the WWARN repository, and standardised using a methodology described in the WWARN Data Management and Statistical Analysis Plan (Text S3) [38].

### Ethical Approval

All data included in this analysis were obtained in accordance with the laws and ethical approvals applicable to the countries in which the studies were conducted, and were obtained with the knowledge and consent of the individual to which they relate. Data were fully anonymised either before or during the process of uploading to the WWARN repository. Ethical approval to conduct individual participant data pooled analyses was granted to WWARN by the Oxford Tropical Research Ethics Committee (OxTREC).

### Dosing Calculation

Where possible, the dose of DHA and PIP administered was calculated from the individual number of tablets administered to each patient daily. Where such data were not available, back-calculations were made, based on the dosing strategy presented in the study protocol, assuming correct adherence to the protocol. Only patients completing a full 3-day treatment regimen and included in the original analysis, were included in the meta-analysis.

### Classification of Study Sites in Transmission Intensity Zones

Study sites were categorised into three strata according to known epidemiology: low, moderate, and high transmission settings. The classification of transmission intensity was based on the author's classification of the site as reported in the study publication. Where no transmission information was reported, then the transmission intensity was defined based on the triangulation of information available from: (i) the study protocol(s), (ii) observed reinfection rate, and (iii) transmission estimates obtained from the Malaria Atlas Project [39]. Further information about this classification is available in the Text S4.

### Statistical Analysis

All statistical analyses were carried out using R (Version 2.14.0, The R Foundation for Statistical Computing), on the basis of an *a priori* statistical plan [40]. The full statistical plan is available in Text S5. The primary endpoint used in this analysis was the PCR-adjusted risk of *P. falciparum* recrudescence at the end of study follow-up. Secondary endpoints included the new infections of *P. falciparum*, parasitological clearance rates, and gametocyte carriage. The incidence risk of these endpoints at day 28, day 42, and day 63 was computed using survival analysis (Kaplan–Meier [K-M] estimates). Definitions of outcome status and censoring are detailed in the WWARN Clinical Module DMSAP v1.2 [38]. The K-M estimates were generated using all the individual patient data and any comparison of K-M survival curves were performed using log rank tests stratified by study and study site. For risk factor analyses, the dose of PIP was considered as a risk factor for recrudescence and reinfection because of its long half-life, whereas the dose of DHA was considered as a risk factor for parasitological clearance rates and gametocyte carriage due to its more rapid anti-parasitic activity and its shorter half-life. Univariable and multivariable analysis of risk factors associated with the primary and secondary endpoints (PCR-adjusted new infection) of interest was conducted using Cox's proportional hazards regression model in a one-step analysis by combining all the individual patient data in a single analysis. In order to account for within study clustering, a shared frailty model was used, in which a random effect was applied to both the study and study site (by combining study and study sites). The overall assumption of proportional hazards was assessed by a global test, and also separately for each of the covariates in the final model. The assumption of proportional hazards was tested for each of the individual studies, although this could not be applied to studies with a low number of events (fewer than ten). All variables significant at 10% level in univariable analysis were included in the multivariable analysis. Inclusion of covariates in the final model was based on their effect on model coefficients and the degree to which they improved the overall model based on a likelihood ratio test. The manufacturer of the drugs used in the studies and the methodology of calculation of mg per kg dose were included as potential covariates.

The population attributable risks (PARs) for treatment failure were calculated based on the prevalence of the risk factor in the population and its associated relative risk (adjusted hazard ratio) [41]. PARs were computed for exposure to a PIP dose below 48 mg/kg (the lower bound of WHO defined therapeutic range for PIP) and for a baseline parasitemia greater than 100,000/µl. The overall PAR (for a combination of risk factors), which is non-additive, was calculated as 1−[(1−PAR_1_)×(1−PAR_2_)×…×(1−PAR_n_)]. In the final multivariable model, the predicted effect of increasing the mg/kg dose of PIP was calculated for every five unit increase, starting from 30 mg/kg. The 95th percentile of predicted risk was computed for each mg/kg dosage of PIP using each patient individual covariates evaluated at average random effect. Although the primary analysis was focussed on risk factors affecting the efficacy of the DP, the relationship between drug dose and gastrointestinal side effects (vomiting and diarrhoea) was also explored using logistic regression with random effects fitted for the individual study and study sites.

## Results

### Characteristics of Included Studies

**Figure 1:**
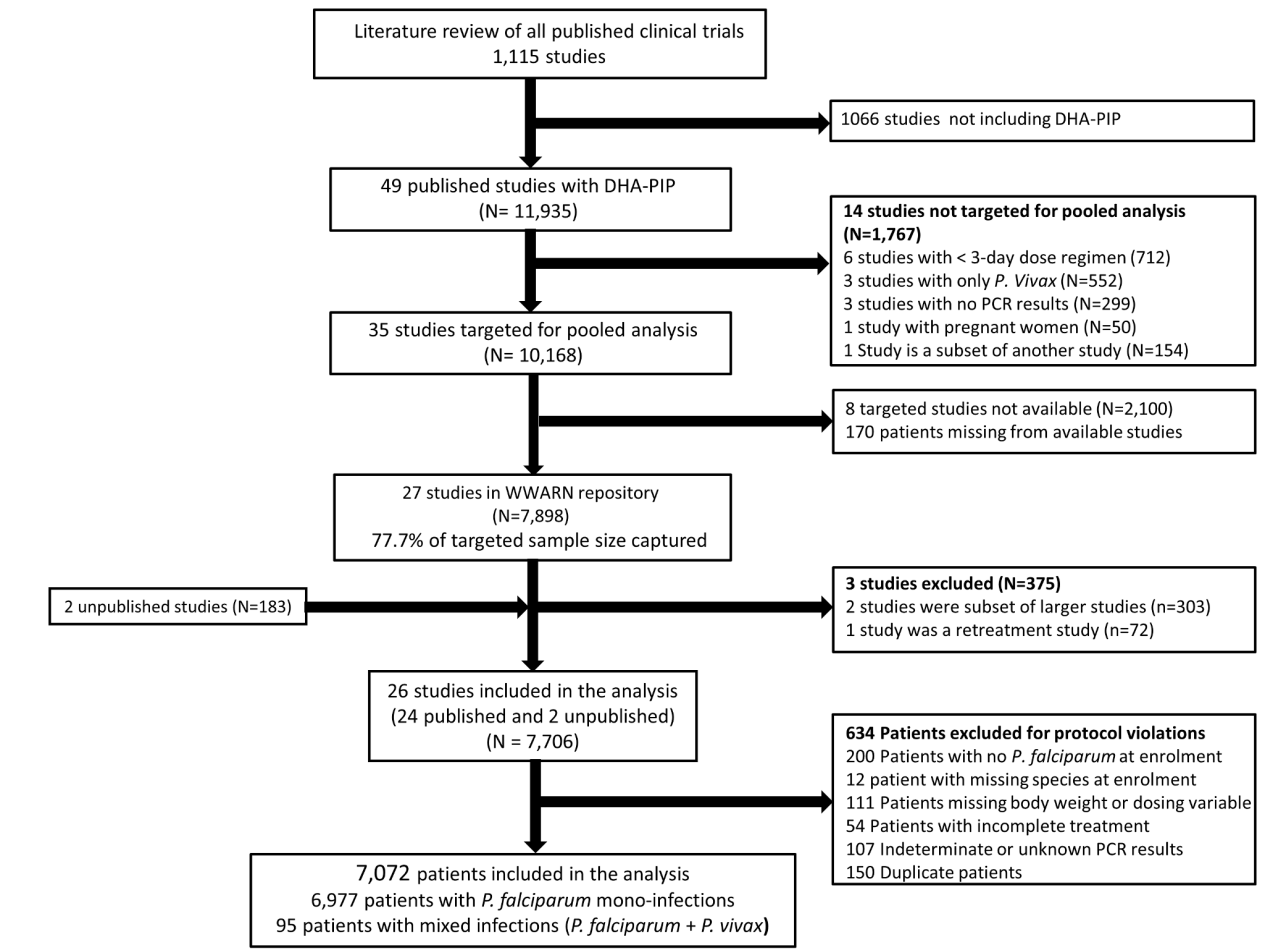
Patient flowchart.

In total, 23 principal investigators (PIs) were approached, of whom 16 submitted data from 29 studies contributing 8,081 patients for the pooled analysis. These data were derived from 77.7% (7,898/10,168) of patients reported in 77.1% (27/35) of the targeted published studies and an additional 183 patients from two unpublished studies (Figure 1). Three of these studies (*n* = 375) did not meet the inclusion criteria and 634 (8.2%) patients from the rest of the studies were excluded for protocol violations. In total 7,072 patients from 26 studies representing 70% of the targeted published literature on this treatment regimen, were included in the final analysis (Figure 1), of whom 2,807 (39.7%) were from 12 studies conducted in Asia, 4,009 (56.7%) from 13 studies from Africa, and 256 (3.6%) from one study conducted in South America (Table 1). Dosing was based on age and weight categories in two studies (*n* = 471), age category in one study (*n* = 124), and on weight bands in the remaining 23 studies (*n* = 6,477). Six studies (*n* = 2,072) followed up patients for 28 days, 12 studies (*n* = 2,664) for 42 days, one study (*n* = 58) for 56 days, and seven studies (*n* = 2,278) for 63 or more days. Three different combinations of DHA and PIP were used in the different studies; 51.4% (*n* = 3,636) of patients were treated with Artekin or Duo-Cotecxin(Holley-Cotec Pharmaceuticals Co), 47.8% (*n* = 3,381) with Eurartesim (Sigma Tau Industrie Farmaceutiche Riunite), and 0.8% (*n* = 55) of the patients were treated with Artecan (OPC Pharmaceutical). Drug intake was reported in all studies, with full supervision in 24 (92.3%) of the studies, partial supervision in one (3.8%), and a combination of full supervision and no supervision in one study (3.8%). Overall 2,628 (37.2%) patients were recruited in areas of high malaria transmission, 1,194 (16.9%) in areas of moderate transmission, and 3,250 (46%) in low transmission areas. Parasite genotyping was carried out in 25 studies: with 17 studies (*n* = 5,751) using three markers (MSP1, MSP2, and GLURP); three studies (*n* = 528) using two markers (MSP1, MSP2); three studies (*n* = 578) using MSP1, MSP2, and microsatellites; one study (*n* = 116) using only microsatellites; and in one study, the genotyping method was not stated (*n* = 41). Genotyping was not carried out in one study (*n* = 58) as there were no recurrent infections.

**Table 1:** Studies included in the meta-analysis.

Study	Year	Region	Country	Number of Patients Treated With DP	Age Range (y Except Where Indicated)	Reference
4ABC Study Group	2011	Africa	Multicentred	1,475	0.5–5	[12]
Adam et al.	2010	Africa	Sudan	75	≥0.5	[17]
Arinaitwe et al.	2009	Africa	Uganda	119	1 m to 1	[18]
Ashley et al.	2004	Asia	Thailand	487	1–65	[19]
Ashley et al.	2005	Asia	Thailand	333	1–65	[20]
Awab et al.	2010	Asia	Afghanistan	59	2–52	Unpublished
Bassat et al.	2009	Africa	Multicentred	1,038	0.5–5	[11]
Borrmann et al.	2011	Africa	Kenya	233	0.5–5	[21]
Gaye et al.	2011	Africa	Senegal	124	6–63	Unpublished
Grande et al.	2007	S. America	Peru	262	5–60	[22]
Hasugian et al.	2007	Asia	Indonesia	168	1–56	[23]
Janssens et al.	2007	Asia	Cambodia	228	2–65	[24]
Kamya et al.	2007	Africa	Uganda	211	0.5–10	[25]
Karema et al.	2006	Africa	Rwanda	252	1–5	[26]
Karunajeewa et al.	2008	Asia	Papua New Guinea	186	0.5–5	[27]
Mayxay et al.	2006	Asia	Laos	110	≥1	[28]
Mens et al.	2008	Africa	Kenya	73	0.5–12	[29]
Ratcliff et al.	2007	Asia	Indonesia	387	1–60	[30]
Sawa et al.	2013	Africa	Kenya	145	0.5–10	[31]
Smithuis et al.	2006	Asia	Myanmar	327	≥1	[32]
Smithuis et al.	2010	Asia	Myanmar	161	≥1	[33]
Tran et al.	2012	Asia	Vietnam	55	>10	[34]
Valecha et al.	2010	Asia	Multicentred	769	3 m–65	[13]
Yavo et al.	2011	Africa	Multicentred	197	2–77	[35]
Yeka et al.	2008	Africa	Uganda	215	0.5–10	[36]
Zongo et al.	2007	Africa	Burkina Faso	187	≥0.5	[37]

Full details of the references and study design are available in Text S1.

### Baseline Characteristics

The baseline characteristics of patients included in the analysis are documented in Table 2. The median age of patients was 4.2 years (range 0.35–75 years), with 6.2% (439/7,072) younger than 1 year, 48.5% (3,429/7,072) from 1 up to 5 years, 13.4% (944/7,072) from 5 up to 12 years, and 31.9% (2,260/7,072) being 12 years or older. Compared to patients from Asia, those from African sites were significantly younger (median: 2.6 years [interquartile range (IQR): 1.5–4, range: 0.35–75] versus median: 18 years [IQR: 8–30, range: 0.7–65], respectively; *p*<0.001) and had a higher median baseline parasitemia (26,520 µl^−1^ [IQR: 8,379–62,400] versus 8,530 µl^−1^ [IQR: 2,240–29,026]; *p*<0.001). In the 256 patients from South America the median age was 23.5 years (IQR: 13–39) with a median baseline parasitemia of 6,274.5 µl^−1^ (IQR: 3,272–9,995).

**Table 2:** Baseline characteristics of patients included in the analysis.

Variable	Asia	Africa	South America^a^	Overall
***n***	2,807 (39.7%)	4,009 (56.7%)	256 (3.6%)	7,072 (100%)
**Study period**	2002–2011	2003–2010	2003–2005	2002–2011
**Gender**				
Female	34.0% [953/2,807]	46.8% [1,875/4,009]	42.2% [108/256]	41.5% [2,936/7,072]
Male	66.1% [1,854/2,807]	50.3% [2,018/4,009]	57.8% [148/256]	56.8% [4,020/7,072]
Missing	0% [0/2,807]	2.9% [116/4,009]	0% [0/256]	1.6% [116/7,072]
**Age**				
Median age [range] in years	18 [0.7–65]	2.6 [0.35–75]	23.5 [5–59]	4.2 [0.35–75]
<1 y	0.2% [5/2,807]	10.8% [434/4,009]	0% [0/256]	6.2% [439/7,072]
1 to <5 y	12.9% [361/2,807]	76.5% [3,068/4,009]	0% [0/256]	48.5% [3,429/7,072]
5 to <12 y	20.9% [587/2,807]	7.8% [312/4,009]	17.6% [45/256]	13.4% [944/7,072]
≥12 y	66.1% [1,854/2,807]	4.9% [195/4,009]	82.4% [211/256]	31.9% [2,260/7,072]
**Body weight**				
Median weight [range] in kg	43 [5–87]	11.6 [2,5–98]	52.5 [1,14–93.5]	14.9 [5–98]
5 to <7 kg	0.2% [6/2,807]	2.8% [111/4,009]	0% [0/256]	1.7% [117/7,072]
7 to <13 kg	11.0% [309/2,807]	58.4% [2,342/4,009]	0% [0/256]	37.5% [2,651/7,072]
13 to <24 kg	19.2% [540/2,807]	31.6% [1,265/4,009]	12.5% [32/256]	26.0% [1,837/7,072]
24 to <36 kg	9.8% [276/2,807]	2.8% [112/4,009]	9.4% [24/256]	5.8% [412/7,072]
36 to <75 kg	59.4% [1,666/2,807]	4.1% [165/4,009]	76.6% [196/256]	28.7% [2027/7,072]
75 to <100 kg	0.4% [10/2,807]	0.3% [14/4,009]	1.6% [4/256]	0.4% [28/7,072]
**Treatment supervision**				
Full	88.7% [2,490/2,807]	93.0% [3,728/4,009]	100% [256/256]	91.5% [6,474/7,072]
Partial	11.3% [317/2,807]	3.9% [157/4,009]	0% [0/256]	6.7% [474/7,072]
Not stated	0% [0/2,807]	3.1% [124/4,009]	0% [0/256]	1.8% [124/7,072]
**Drug formulations** b				
Eurartesim	27.0% [759/2,807]	65.4% [2,622/4,009]	0% [0/256]	47.8% [3,381/7,072]
Artekin	59.3% [1,664/2,807]	6.2% [249/4,009]	100% [256/256]	30.7% [2,169/7,072]
Duo-Cotecxin	11.7% [329/2,807]	28.4% [1,138/4,009]	0% [0/256]	20.7% [1,467/7,072]
Artecan	2.0% [55/2,807]	0% [0/4,009]	0% [0/256]	0.8% [55/7,072]
**Enrolment clinical parameters**				
Median parasitemia [IQR]	8,530 [2,240.5–29,026.8]	26,520 [8,739–62,400]	6,274.5 [3,272–9,995]	16,580 [4,782–473,000]
Mixed infections with *P. vivax*	3.4% [95/2,807]	0% [0/4,009]	0% [0/256]	1.3% [95/7,072]
Haemoglobin [mean ± SD]	11.2±2.6	9.4±1.8	12.2±1.7	10.2±2.3
Anemic (Hb <10 g/dl)	29.9% [765/2,558]	61.8% [2,247/3,639]	7.4% [19/256]	47.0% [3,031/6,453]
Gametocyte carriage	19.1% [291/1,527]	9.7% [361/3,713]	12.5% [32/256]	12.5% [684/5,496]
Fever (>37.5°C)	49.2% [1,197/2,433]	61.5% [2,421/3,938]	46.1% [118/256]	56.4% [3,736/6,627]

^a^ Data from one study conducted in Peru.

+ 320 mg PIP or 20 mg DHA + 160 mg PIP in paediatric formulation (full details are given in Text S1).^b^ DHA-PIP tablets strength was 40 mg DHA

### Distribution of Dihydroartemisinin Dosing

The median total dose of DHA administered was 6.8 mg/kg [IQR: 6–8, range: 2.3–22.9] (Table 3). Overall 19.6% (1,387/7,072) of the patients received a total dose of DHA less than 6 mg/kg (the lower limit for DHA recommended by the WHO), although this varied significantly between age groups. None of the patients were exposed to a total DHA dose greater than 30 mg/kg (the upper limit recommended by the WHO). In a multivariable analysis controlling for weight, children from 1 up to 5 years were at the greatest risk of being exposed to a total dose of DHA < 6 mg/kg compared to infants younger than 1 year (adjusted odds ratio [AOR] = 1.7 [95% CI 1.2–2.3; *p* = 0.002]), children from 5 up to 12 years (AOR = 6.6 [95% CI 4.4–4.9; *p*<0.001]) and 12 years or older (AOR = 72.9 [95% CI 37.3–142.5; *p*<0.001]); *p*<0.005 for all comparisons. Patients from African sites were at greater risk of being exposed to a total dose of DHA dose below 6 mg/kg, compared to those from Asia (odds ratio [OR] = 3.3 [95% CI 1.5–7.6; *p* = 0.004]), but, this was no longer significant after adjusting for the age and weight of the patient (AOR = 2.13 [95% CI 0.90–5.07; *p* = 0.09]).

**Table 3:** Total piperaquine and dihydroartemisinin dose by age and weight categories.

Characteristics	*n*	PIP Dosage (mg/kg)		DHA Dosage (mg/kg)		Exposure to Dose Outside WHO Range		
		Median [IQR]	Range	Median [IQR]	Range	PIP Dose <48 mg/kg	PIP Dose >78 mg/kg	DHA <6 mg/kg^a^
**Overall**	7,072	53.3 [48–62.6]	18.2–182.9	6.8 [6.0–8.0]	2.3–22.9	20.3% [1,437/7,072]	2.2% [155/7,072]	19.6% [1,387/7,072]
**Age category**								
<1 y	439	60 [53.3–67.61]	34.8–80.0	7.5 [6.7–8.5]	4.4–10.0	12.5% [55/439]	5.7% [25/439]	12.5% [55/439]
1 to <5 y	3,429	53.3 [45.7–64.0]	22.5–96.0	6.7 [5.7–8.0]	2.8–13.9	28.6% [979/3,429]	0.6% [20/3,429]	28.5% [976/3,429]
5 to <12 y	944	57.1 [51.2–65.4]	25.3–182.9	7.2 [6.4–8.4]	3.2–22.9	9.5% [90/944]	8.3% [78/944]	8.3% [78/944]
≥12 y	2,260	53.3 [49.7–58.2]	18.2–151.6	6.7 [6.3–7.5]	2.3–18.9	13.9% [313/2,260]	1.4% [32/2,260]	12.3% [278/2,260]
**Weight category**								
5 to <7 kg	117	42.9 [37.5–77.4]	34.8–80	5.4 [4.7–9.7]	4.4–10	53.9% [63/117]	23.9% [28/117]	53.9% [63/117]
7 to <13 kg	2,651	48.4 [43.6–58.5]	22.5–182.9	6.1 [5.5–7.4]	2.8–22.9	36.2% [960/2,651]	1.1% [30/2,651]	36.2% [960/2,651]
13 to <24 kg	1,837	64.0 [55.4–68.6]	25.3–151.6	8.0 [6.9–8.6]	3.2–18.9	5.1% [94/1,837]	3.5% [64/1,837]	4.4% [80/1,837]
24 to <36 kg	412	57.7 [53.3–66.2]	38.8–98.5	7.3 [6.8–8.6]	4.9–13.9	2.4% [10/412]	5.3% [22/412]	2.4% [10/412]
36 to <75 kg	2,027	53.3 [49.2–57.6]	18.2–80.0	6.7 [6.2–7.4]	2.3–13.3	14.3% [290/2,027]	0.5% [11/2,027]	12.5% [254/2,027]
75 to <100 kg	28	37.4 [32.8–48.3]	26.1–56.5	4.7 [4.1–6.04]	3.3–7.1	71.4% [20/28]	0% [0/28]	71.4% [20/28]
**Region**								
Asia	2,807	55.4 [50.5–62.6]	18.2–182.9	7.1 [6.4–8.0]	2.2–22.9	11.4% [320/2,807]	3.7% [104/2,807]	9.6% [270/2,807]
Africa	4,009	53.3 [46.2–64.0]	25.3–96.0	6.7 [5.8–8.0]	3.2–12	27.5% [1,103/4,009]	1.3% [51/4,009]	27.51% [1,103/4,009]
S. America	256	52.1 [50.1–54.9]	46.6–68.6	6.5 [6.3–6.9]	5.8–8.57	5.5% [14/256]	0% [0/256]	5.5% [14/256]

[5]. No patient was exposed to a DHA dose >30 mg/kg.^a^ The WHO therapeutic guidelines recommend a target dose for PIP of 54 mg/kg over 3 days with a range from 48 to 78 mg/kg; and a target dose for DHA of 12 mg/kg over 3 days with a range from 6 to 30 mg/kg

### Distribution of Piperaquine Dosing

**Figure 2:**
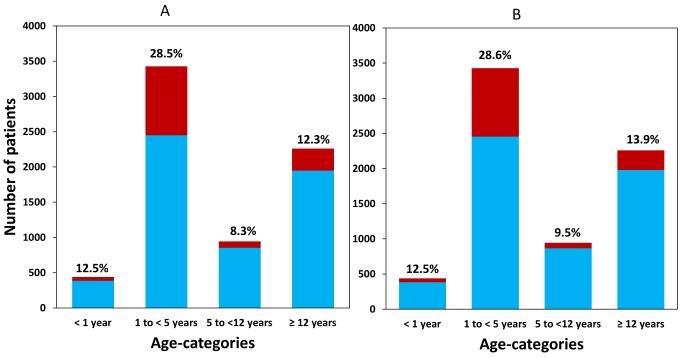
Available patient data within each age category for (A) dihydroartemisinin and (B) piperaquine. The patients receiving a total mg/kg dose below the WHO therapeutic range (6 mg/kg and 48 mg/kg, respectively) are shown in dark columns and as a percentage of all patients on top of the bar.

The median total dose of PIP administered was 53.3 mg/kg (IQR: 48.0–62.6, range: 18.2–182.9 mg/kg), but this varied significantly between age groups (Table 3). Overall, 20.3% (1,437/7,072) of patients received a total dose of PIP below 48 mg/kg (the lower limit recommended by WHO) (Figure 2), whereas only 2.2% (155/7,072) received a dose greater than 78 mg/kg (the WHO upper limit). Young children (from 1 up to 5 years of age) were at greater risk of receiving a total dose of PIP below 48 mg/kg compared to infants <1 year (OR = 2.3 [95% CI 1.7–3.2; *p*<0.001]), children from 5 up to 12 years (OR = 2.9 [95% CI 2.0–4.2; *p*<0.001]), and patients 12 years or older (OR = 2.3 [95% CI 1.7–3.3; *p*<0.001]). These comparisons remained statistically significant after adjusting for body weight (*p*<0.005 for all comparisons). Patients from African sites were at greater risk of being exposed to a total dose of PIP below 48 mg/kg, compared to those from Asia (OR = 3.0 [95% CI 1.3–7.0; *p* = 0.01]), however like DHA, this was no longer significant after adjusting for age. There was no significant difference in the risk of under dosing PIP between patients from South America and Asia.

### Early Parasitological Response

The overall speed of parasite clearance was rapid (Table 4), with the overall parasite positivity rate (PPR) decreasing from 59% (3,083/5,222) on day 1, to 9.1% (576/6,321) on day 2, and 1.2% (70/5,697) on day 3. The PPR on day 1 and day 2 was higher in children younger than 5 years compared to older children and adults (≥5 years), with this difference being greatest on day 1 (OR = 1.70 [95% CI 120–2.40; *p* = 0.002]).

**Table 4:** Parasite positivity rates on days 1, 2, and 3.

Characteristics	All Patients	Day 1		Day 2		Day 3	
Age category	*n*	Positive	PPR [95% CI]^a^	Positive	PPR [95%CI]^a^	Positive	PPR [95% CI]^a^
<1 y	439	144/226	63.7 [48.8–78.6]	27/433	6.2 [2–10.5]	3/432	0.7 [0–1.5]
1 to <5 y	3,429	1,610/2,522	63.8 [54.1–73.6]	283/3,153	9 [3.1–14.8]	26/3,061	0.8 [0–1.7]
5 to <12 y	944	303/541	56 [40.7–71.4]	39/713	5.5 [1.1–9.8]	8/559	1.4 [0–2.9]
≥12 y	2,260	1,026/1,933	53.1 [42.5–63.6]	227/2,022	11.2 [4.3–18.2]	33/1,645	2 [0.5–3.5]
**DHA dose (mg/kg)**							
<6 mg/kg	1,387	718/1,137	63.1 [53.6–72.7]	140/1,297	10.8 [4.7–16.8]	18/1,212	1.5 [0.5–2.5]
≥6 mg/kg	5,685	2,365/4,085	57.9 [50–65.8]	436/5,024	8.7 [4.7–12.7]	52/4,485	1.2 [0.4–1.9]
**PQP dose (mg/kg)**							
<48 mg/kg	1,437	750/1,185	63.3 [54.1–72.4]	144/1,345	10.7 [4.9–16.5]	19/1,257	1.5 [0.5–2.5]
≥48 mg/kg	5,635	2,333/4,037	57.8 [49.8–65.8]	432/4,976	8.7 [4.6–12.7]	51/4,440	1.1 [0.4–1.9]
**Region**							
Asia	2,807	1,316/2,238	58.8 [47.5–70.1]	266/2,160	12.3 [5.7–18.9]	40/1,546	2.6 [1.2–4]
Africa	4,009	1,685/2,730	61.7 [51.5–72]	307/3,907	7.9 [2.6–13.1]	30/3,897	0.8 [0–1.5]
S. America	256	3/254	32.3 [26.8–38.3]	3/254	1.2 [0.4–3.4]	0/354	0 [0–1.5]
**Overall**	7,072	3,083/5,222	59 [51.3–66.8]	576/6,321	9.1 [5–13.3]	70/5,697	1.2 [0.5–2]

^a^ Parasite positivity rates were calculated from those with in who a blood film was taken on that day; cases without a smear were removed from the denominator.

The dose of DP was a significant predictor of parasite positivity on day 3, with an OR of 0.81 (95% CI 0.67–0.97, *p* = 0.022) per unit increase in mg/kg of DHA and 0.97 (95% CI 0.94–0.99; *p* = 0.026) per unit increase in mg/kg PIP dose. These ORs remained statistically significant after controlling for age, baseline parasitemia, and transmission setting, AOR = 0.80 (95% CI 0.66–0.96; *p* = 0.017) and 0.97 (95% CI 0.94–0.99; *p* = 0.020) per unit increase in mg/kg DHA and PIP, respectively. The dose of DP was not a significant predictor of parasite positivity on day 1 or day 2.

### Late Parasitological Response

**Figure 3:**
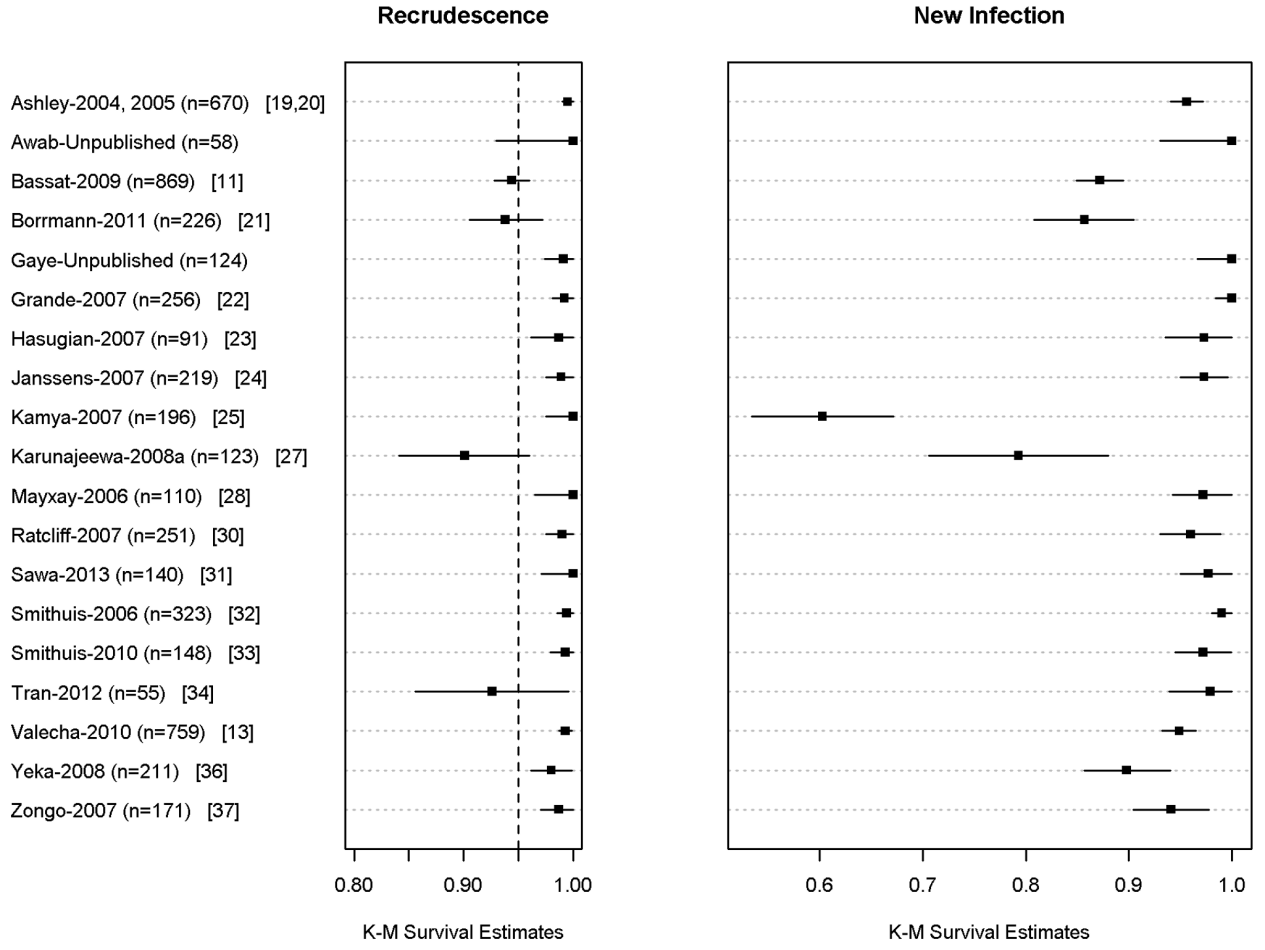
PCR adjusted risk of recrudescent and new infections at day 42 for individual studies. The full citations for these studies are available in Text S1. The figure excludes data from 6 studies in which active follow-up was stopped at Day 28 [12,17,18,26,29,36]. The results for Ashley-2004 [19] and Ashley-2005 [20] are presented pooled since the datasets did not distinguish between the studies.

In total, 704 (9.9%) patients had recurrent parasitemia detected during follow-up, of whom 136 (1.9%, 136/7,072) were confirmed by PCR as true recrudescence. In seven studies with a follow-up duration of 63 days (*n* = 2,278 and 34 confirmed recrudescent failures), nine (26.5%) patients failed before day 28, 16 (47%) patients failed between day 28 and day 42, and nine (26.5%) patients recurred after day 42. Overall PCR-corrected K-M survival estimates were 98.8% (95% CI 98.5–99%) at day 28, 97.7% (95% CI 97.3–98.1%) at day 42, and 97.2% (95% CI 96.7–97.7%) at day 63 (Figure 3; Table 5). The corresponding figures in children from 1 up to 5 years of age were 97.9% (95% CI 97.4–98.4%) at day 28, 95.8% (95% CI 94.9–96.7%) at day 42, and 94.4% (95% CI 92.6–96.2%) at day 63.

**Table 5:** PCR-corrected adequate clinical and parasitological response of dihydroartemisinin-piperaquine by major categories.

Kaplan–Meier survival estimates^a^			
Characteristics	Day 28 [95% CI]	Day 42 [95% CI]	Day 63 [95% CI]
	At risk: 6,534	At risk: 4,238	At risk: 1,569
**Gender**			
Male	98.8 [98.4–99.1]	97.5 [97–98.1]	97 [96.3–97.7]
Female	98.7 [98.2–99.1]	97.9 [97.4–98.5]	97.5 [96.7–98.3]
**Age category**			
<1 y	99.5 [98.9–100]	97.3 [95.3–99.3]	97.3 [95.3–99.3]
1 to <5 y	97.9 [97.4–98.4]	95.8 [94.9–96.7]	94.4 [92.6–96.2]
5 to <12 y	99.3 [98.8–99.9]	99.1 [98.5–99.7]	99.1 [98.5–99.7]
≥12 y	99.6 [99.4–99.9]	99.2 [98.8–99.6]	98.7 [98.2–99.3]
**Region**			
Asia	99.2 [98.9–99.6]	98.9 [98.4–99.3]	98.4 [97.9–98.9]
Africa	98.4 [98–98.8]	96.3 [95.5–97]	94.3 [92–96.6]
S. America^b^	99.2 [98.0–100.0]	99.2 [98.0–100.0]	99.2 [98.0–100.0]
**Overall**	98.8 [98.5–99]	97.7 [97.3–98.1]	97.2 [96.7–97.7]

–Meier estimates were generated using all the individual data rather than combining estimates from individual trials.^a^ Kaplan

^b^ One study from Peru with no failures after day 25.

### Risk Factors for Recrudescence

Six univariate factors on admission were associated with recrudescent parasitemia by day 42: the mg/kg PIP dose (hazard ratio [HR] = 0.86 [95% CI 0.78–0.94; *p* = 0.001] for every 5 mg/kg increase), baseline parasitemia (log scale) (HR = 1.26 [95% CI 1.10–1.44; *p* = 0.001]), presence of gametocytes at baseline (HR = 1.79 [95% CI 1.05–3.04; *p* = 0.032]), being from 1 up to 5 years of age (HR = 3.71 [95% CI 1.66–8.26; *p* = 0.002]), admission haemoglobin (HR = 0.92 [95% CI:0.83–1.01; *p* = 0.080]), and body weight (HR = 0.97 [95% CI 0.95–0.99; *p* = 0.010]) (Table 6). In a multivariable model, after adjusting for age and parasitemia the dose of PIP was a significant predictor for recrudescence (adjusted hazard ratio [AHR] = 0.87 [95% CI 0.79–0.95; *p* = 0.002] for every 5 mg/kg increase in PIP dose). In total 3% (44/1,437) of patients exposed to a dose of PIP less than 48 mg/kg experienced a recrudescent infection compared to 1.5% (83/5,633) of those who received a dose greater than 48 mg/kg kg (HR = 1.48 [95% CI 0.99–2.19; *p* = 0.05]).

**Table 6:** Univariate and multivariate risk factors for PCR confirmed recrudescent failures at day 42.

Variable	Total *n* [*n*]^a^	Univariable Analysis		Multivariable Analysis^b^		PAR^c^	
		Crude HR [95% CI]	*p*-Value	Adjusted HR [95% CI]	*p*-Value	Freq.	PAR
Age (y)	7,070 [127]	0.97 [0.94–1]	0.037	—	—	—	—
Body weight (kg)	7,070 [127]	0.97 [0.95–0.99]	0.010	—	—	—	—
PIP dose (mg/kg) (every 5 unit increase)	7,070 [127]	0.86 [0.78–0.94]	0.001	0.87 [0.79–0.95]	0.002	20.3%^d^	7.7%
**Enrolment clinical variables**							
Parasitemia (log-scale)	7,070 [127]	1.26 [1.10–1.44]	0.001	1.23 [1.08–1.41]	0.003	9.3%	6.5%
Baseline fever (>37.5°C)	6,625 [124]	1.08 [0.75–1.56]	0.670	—	—	—	—
Baseline haemoglobin (g/dl)	6,670 [122]	0.92 [0.83–1.01]	0.080	—	—	—	—
Baseline anaemia (hb <10 g/dl)	6,670 [122]	1.22 [0.81–1.84]	0.350	—	—	—	—
Baseline gametocyte carriage	5,494 [111]	1.79 [1.05–3.04]	0.032	—	—	—	—
**Gender**							
Female (reference)	2,935 [49]	1	—	—	—	—	—
Male	4,019 [78]	1.35 [0.94–1.94]	0.100	—	—	—	—
**Age category**							
≥12 y (reference)	2,259 [15]	1	—	—	—	—	—
<1 y	439 [7]	2.36 [0.79–7.06]	0.200	2.39 [0.79–7.25]	0.120	6.2%	7.8%
1 to <5 y	3,429 [9]	3.71 [1.66–8.26]	0.002	3.22 [1.42–7.33]	0.005	48.5%	53.5%
5 to <12 y	943 [7]	1.48 [0.56–3.91]	0.610	1.56 [0.59–4.13]	0.370	13.3%	5.7%
**Region**							
Asia (reference)	2,805 [28]	1	—	—	—	—	—
Africa	4,009 [97]	1.74 [0.67–4.51]	0.260	—	—	—	—
S. America	256 [2]	0.45 [0.02–8.61]	0.600	—	—	—	—
**Treatment supervision**							
Full (reference)	6,472 [124]	1	—	—	—	—	—
Partial	474 [2]	0.26 [0.04–1.73]	0.170	—	—	—	—
**Co-administration with fat**							
With fat meal (reference)	960 [16]	1	—	—	—	—	—
Without fat meal	2,448 [77]	2.92 [0.73–11.55]	0.130	—	—	—	—
Unknown	3,662 [34]	0.95 [0.25–3.55]	0.940	—	—	—	—
**Drug Formulation**							
Duo-Cotecxin (reference)	1,467 [18]	1	—	—	—	—	—
Artekin	54 [3]	2.92 [0.14–58.56]	0.480	—	—	—	—
Artecan	2,168 [22]	0.96 [0.27–3.46]	0.960	—	—	—	—
Eurartesim	3,381 [84]	1.47 [0.48–4.50]	0.500	—	—	—	—

*n*) for each variable/levels of factor with number of recrudescence [*n*] by day 42.^a^ Number of patients (

*p* = 0.32 for global test for proportional hazards assumption. Variance of random effect  = 1.17. Non-significant likelihood ratio test for weight (*p* = 0.27) and hemoglobin (*p* = 0.26) and thus dropped from the multivariable analysis. Baseline gametocytemia (*p* = 0.02) improved the model but 22.3% (1,576/7,070) of patient had missing observation for this variable and hence not kept for multivariable analysis. Inclusion (or exclusion) of gametocytemia didn't alter the significance of the other variable and its effect on model coefficient for age and dose was small.^b^

c Overall PAR for model: 65.1%.

d HR (95% CI) = 1.48 [0.99–2.19] *p* = 0.054 and AHR (95% CI) = 1.39 [0.94–2.06], *p* = 0.10 for mg/kg PIP dose <48 mg/kg in univariable and multivariable analysis, respectively.

Overall, the risk of recrudescence was greatest in the 1 up to 5 year age group, rising to 5.6% (95% CI 3.8–7.4) by day 63 (Tables 5 and 6). Compared to patients older than 12 years, children from 1 up to 5 years of age had HR of 3.71 (95% CI 1.66–8.26; *p* = 0.002) for the risk of recrudescence. The risk remained significant after adjusting for baseline parasitemia, and the mg/kg dose of PIP (AHR = 3.22 [95% CI 1.42–7.33; *p* = 0.005]). The population attributable risks of recrudescence are presented in Table 6. Overall, the model accounted for 65.1% of all treatment failures, with a low dose of PIP accounting for 7.7% and children from 1 up to 5 years of age for 53.5%.

**Figure 4:**
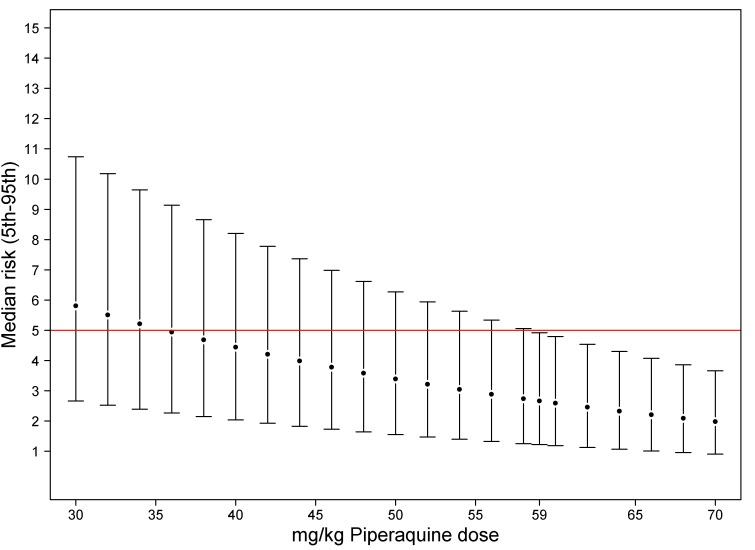
Percentiles of predicted risk [5th-median-95th] of recrudescent failure at day 42 in children aged from 1 up to 5 years computed from multivariate model. Risk was calculated for each individual using their own values. The error bars show the 5th and 95th percentiles of predicted risk of recrudescence failure.

**Figure 5:**
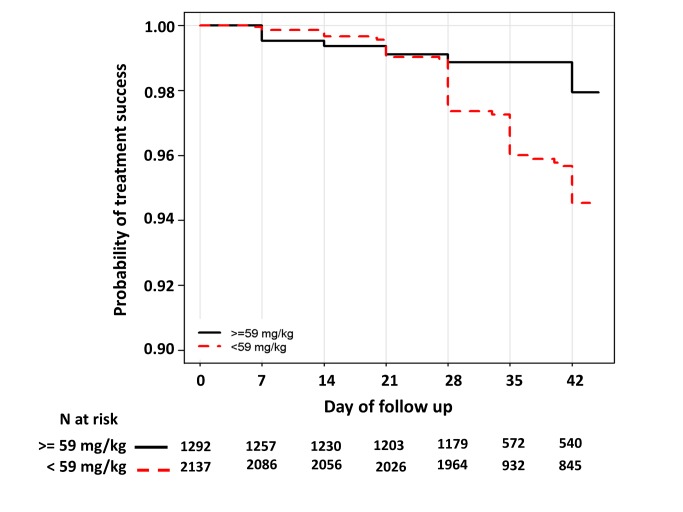
Kaplan–Meier curve for PCR-confirmed recrudescence for children from 1 up to 5 years of age exposed to a dose below or above 59 mg/kg. Log rank test stratified by study sites *p*<0.001. The HR for exposure to a PIP dose below 59 mg/kg was 2.36 (95% CI 1.42–3.91), *p*<0.001 and the AHR 2.03 (95% CI 1.20–3.43), *p* = 0.008; after controlling for parasitemia and body weight.

In children aged from 1 up to 5 years of age (*n* = 3,429, 98 recrudescent failures), five risk factors on admission were associated with recrudescence failure by day 42; age (years) (HR = 0.73 [95% CI 0.59–0.89; *p* = 0.002]), body weight (HR = 0.87 [95% CI 0.79–0.96; *p* = 0.006]), enrolment parasitemia (log-scale) (HR = 1.22 [95% CI 1.04–1.43; *p* = 0.015]), admission haemoglobin (HR = 0.90 [95% CI 0.80–1.01; *p* = 0.079]), and mg/kg PIP dose (HR = 0.86 [95% CI 0.77–0.95; *p* = 0.003] for every 5 unit mg/kg). Since weight and age were collinear only weight was included in the multivariable analysis. In the multivariable model, the dose of PIP remained a significant risk factor (AHR = 0.87 [95% CI 0.78–0.97, *p* = 0.013] for every 5 unit mg/kg). The median total dose of PIP was 48.0 mg/kg (IQR: 42.8–53.3 mg/kg) in young children with recrudescence compared to 53.3 mg/kg (IQR: 45.7–64.0 mg/kg) in those who were cured (*p*<0.001). In the multivariable model every 5 unit increase in mg/kg dose of PIP was associated with a 13% (95% CI 3–22%) decrease in risk of recrudesecence; *p* = 0.013. In a predicted risk model, generated from patients' individual covariates, a dose of 59 mg/kg PIP was sufficient to ensure a day 42 cure rate above 95% (Figure 4). Children aged from 1 up to 5 years receiving a PIP dose below 59 mg/kg were at twice the risk of recrudescence (AHR = 2.03 [95% CI 1.2–3.42; *p* = 0.008]), and this accounted for 39.3% (PAR) of all recrudescent infections. By day 42 the risk of recrudescence in patients receiving a PIP dose below this threshold was 5.5% (95% CI 4.2–6.7) compared to 2.1% (95%: 1.1–3.0) in patients receiving a higher dose, *p*<0.001 (Figure 5).

### Gametocyte Carriage

**Figure 6:**
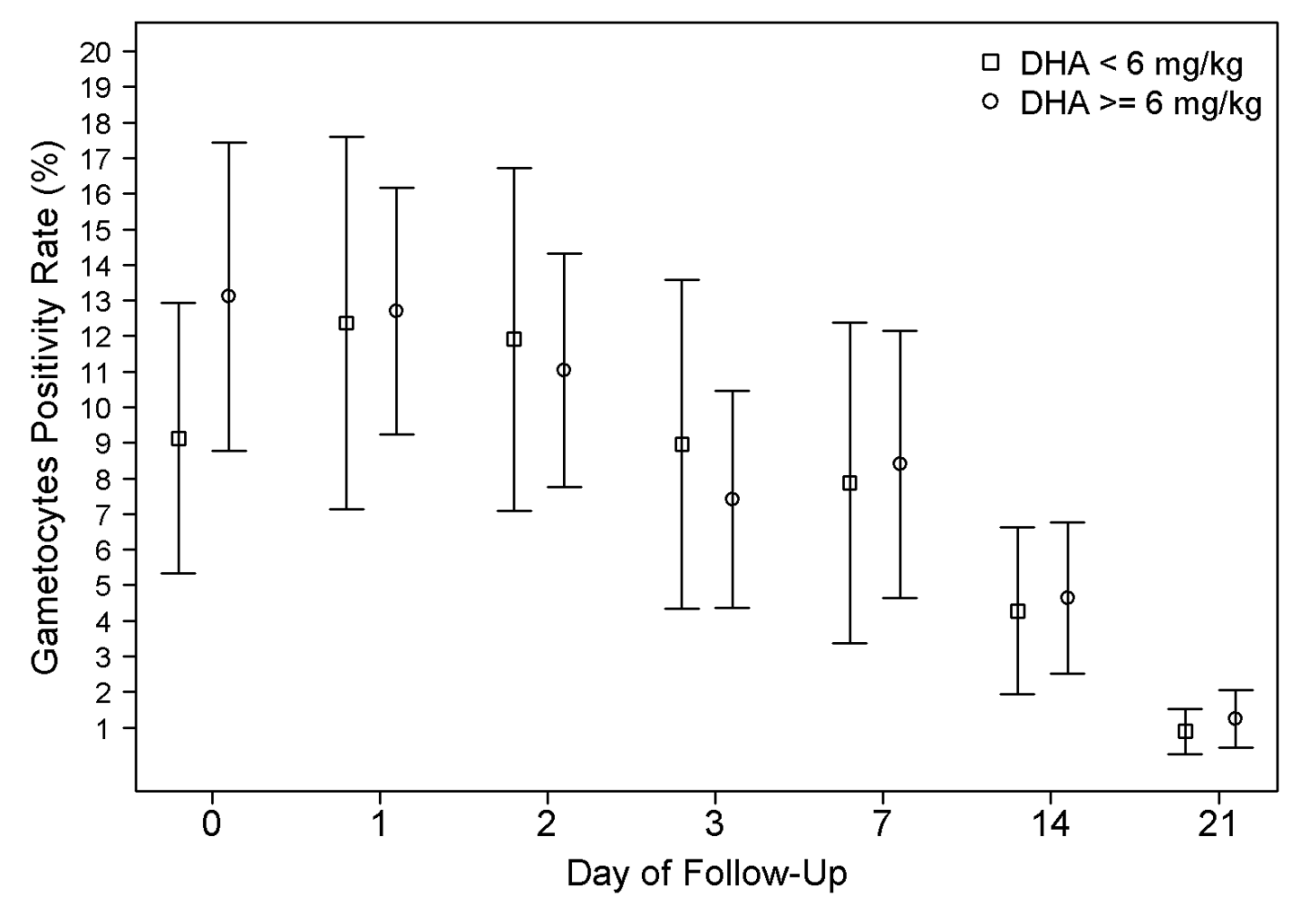
Gametocyte positivity rate (GPR) during follow-up. The error bars show the 95% confidence interval for the positivity rates. The proportions are unadjusted for age and baseline parasitemia. GPR on admission was significantly higher in patients receiving DHA dose ≥6 mg/kg group (*p*<0.001).

The overall gametocyte carriage decreased from 12.3% at enrolment to 8.3% on day 7 a reduction of 4.0% (95% CI 2.8%–5.2%) and fell further thereafter; *p*<0.001 (Figure 6). Exposure to a total DHA dose below 6 mg/kg was associated with a non-significant increased risk of gametocyte carriage on day 7 (OR = 1.34 [95% CI 0.97–1.85; *p* = 0.076]); however, this was significant after adjusting for age, baseline gametocytemia, and parasitemia (AOR = 1.56 [95% CI 1.08–2.24; *p* = 0.015]). The dose of DHA did not correlate with the risk of gametocyte reappearing.

### Safety Parameters

In total 3.2% (54/1,669) of patients with full documentation of tablet administration did not complete a full course of DP. In four (7.4%) cases this was due to adverse events (two patients had recurrent vomiting, one had diarrhoea, and one patient developed a rash). Information regarding acute vomiting of medication was available for ten studies with 8.8% (376/4,272) of the patients vomiting within an hour of drug administration, this proportion being greatest in infants and children from 1 up to 5 years of age (Table 7). Data on patients vomiting within the preceding 24 hours were available in 13 studies and reported in 16.6% (736/4,440) of the patients. The incidence of diarrhoea within the first 7 days of follow-up was available in 11 studies, and occurred in 11.6% (530/4,560) of patients. After controlling for age, parasitemia, and fever at presentation the risks of vomiting or diarrhoea were not correlated with the mg/kg dose of PIP.

**Table 7:** Gastrointestinal adverse events.

Characteristics	All Patients			Patients Aged 1 up to 5 Years Old		
	Acute Vomiting Drug^a^	History of Vomiting in First 3 Days^b^	Diarrhoea in First 7 Days^c^	Acute Vomiting Drug^a^	History of Vomiting in First 3 Days^b^	Diarrhoea in First 7 Days^c^
**Male**	7.8% (196/2,512)	16.7% (421/2,526)	11.3% (301/2,665)	10.8% (140/1,295)	10.8% (151/1,398)	7.7% (97/1,253)
**Female**	10.2% (180/1,760)	16.5% (315/1,914)	12.1% (229/1,895)	12% (142/1,188)	10.0% (130/1,302)	8.5% (96/1,130)
**Age category**						
**<1 y**	30.1% (59/196)	18.2% (48/264)	16% (40/250)	—	—	—
**1 to <5 y**	11.4% (282/2,483)	10.4% (281/2,700)	8.1% (193/2,383)	11.4% (282/2,483)	10.4% (281/2,700)	8.1% (193/2,383)
**5 to <12 y**	5.2% (13/251)	30% (100/333)	15.8% (56/354)	—	—	—
**≥12 y**	1.6% (22/1,342)	26.9% (307/1,143)	15.3% (241/1,573)	—	—	—
**PIP dose Category (mg/kg)**						
**<40**	6.6% (13/196)	11.2% (21/187)	9.6% (18/187)	4% (6/1,50)	10.2% (15/147)	6.6% (9/137)
**40 to <45**	10.8% (66/611)	9.6% (58/604)	8.7% (50/576)	11.6% (64/554)	8.0% (45/561)	9% (46/509)
**45 to <50**	7.7% (55/715)	14.4% (106/738)	15.6% (116/743)	11.1% (49/442)	9.7% (47/486)	10.2% (41/403)
**50 to <55**	6.7% (55/823)	19.8% (178/898)	13% (136/1,049)	13% (41/315)	10.6% (38/357)	8.1% (28/345)
**55 to <60**	6.4% (32/501)	22.4% (117/522)	14% (78/558)	13.8% (20/145)	16.5% (29/176)	11.1% (16/144)
**60 to <65**	11.6% (72/621)	17% (117/688)	9.1% (60/661)	12.8% (43/337)	11.1% (45/407)	5.5% (19/347)
**65 to <70**	10.5% (45/427)	18% (81/450)	10.3% (45/436)	11.1% (30/271)	12.3% (36/293)	6.9% (18/259)
**70 to <75**	9.9% (33/334)	13.7% (43/313)	7.9% (24/302)	10.8% (29/268)	9.7% (26/269)	6.8% (16/237)
**75 to <80**	16.7% (4/24)	52.9% (9/17)	4.8% (1/21)	0% (0/1)	0% (0/1)	0% (0/2)
**≥80**	5% (1/20)	26.1% (6/23)	7.4% (2/27)	—	0% (0/3)	—
**Overall**	8.8% (376/4,272)	16.6% (736/4,440)	11.6% (530/4,560)	11.4% (282/2,483)	10.4% (281/2,700)	8.1% (193/2,383)

a At least one episode of vomiting PIP dose within an hour of treatment on day 0, day 1, and day 2.

b At least one episode of vomiting on any days between day 0–day 3.

c At least one episode of diarrhea on any days between day 0–day 7.

A total of 21 severe adverse events (SAEs) were reported from five studies (*n* = 2,873), but in none of the patients in eight other studies (*n* = 1,120). In the remaining 13 studies, severe adverse events were not reported or the data were unavailable. These severe events included one patient with diarrheal disease, considered unrelated to treatment who died, three patients with severe anaemia, three with recurrent vomiting, two with pyomyositis, two with acute pyelonephritis, two with recurrent *P. falciparum* infections, and one patient each with one of the following: abnormal skin, a swollen arm, anorexia, aspiration pneumonia, cerebritis, convulsions, high fever, and hyperparasitemia *P. falciparum* infection. Five patients were younger than 1 year, five were from 1 up to 5 years of age, and eleven were older than 5 years. There was no evidence of these SAEs being related with the mg/kg dose of PIP.

## Discussion

Over the last decade, a number of clinical trials have highlighted the impressive antimalarial efficacy of DP, which was added to the WHO list of antimalarials recommended for first line therapy in 2010 [5]. We present the largest pooled analysis of DP efficacy yet described, to our knowledge, including more than two-thirds of all data from the published literature. Overall the efficacy of DP was excellent, exceeding 98% at day 28 and 97% at day 42, consistent with published results from Africa [12],[17],[18],[21],[25],[26],[29],[31],[35]–[37], Asia [13],[19],[20],[23],[24],[27],[28],[30],[32]–[34], and South America [22]. However our study reveals an important sub-group of patients who are at particular risk of treatment failure.

ACTs have become a cornerstone of the current global strategy for the control and elimination of malaria and are considered a major factor in achieving the substantial gains in malaria control observed in many endemic settings over the past decade. However, these gains are now under threat from the emergence of antimalarial drug resistance, both to the artemisinin derivatives [42],[43] and their partner drugs [44]. It is crucial that current treatment guidelines advocate that drug regimens be deployed using optimal dosing strategies to maximise the likelihood of rapid clinical and parasitological cure, minimize transmission, and retard the onset and spread of drug resistance.

Ideally, antimicrobial dose recommendations should be derived from an understanding of the dose response curve, the age stratified pharmacokinetic profile of the drugs, and awareness of any dose related toxicity. The current WHO guidelines for DP recommend a target PIP dose range between 48 and 78 mg/kg [5]. However these initial recommendations were based predominantly on pharmacokinetic studies in older children and adults [45]. Our meta-analysis of a large and diverse population of patients provides the power to define the dose effect of mg/kg dose on DP therapeutic efficacy across a wide age range and transmission settings.

Compared to adults, children from 1 up to 5 years were at almost 4-fold greater risk of treatment failure (Table 6), with 5.6% of young children at a risk of suffering recrudescent infection by day 63 compared to 2.8% in the overall population. The increased risk in young children has been attributed to reduced host immunity [2], exacerbated by these pauci-immune individuals being at greater risk of presenting with a higher baseline parasitemia, itself an independent risk factor for treatment failure [46],[47]. As predicted, both age and parasitemia were important determinants of DP efficacy. After controlling for these factors, the mg/kg dose of PIP administered was the most important risk factor predictive of treatment failure. There was no difference in efficacy by manufacturer after adjusting for these covariates. Although the 94% efficacy in young children was above the 90% limit at which the WHO recommends a change in treatment policy, it suggests that the currently recommended target dose for PIP in young children is at a critical part of the dose response curve exposing parasites to a strong selective drug pressure [7],[48]. Given the high burden of malaria carried by children between 1 and 5 years this represents a potentially important parasite reservoir that could drive the evolution of PIP resistance and ultimately the decline in DP's efficacy. Our analysis predicts that raising the target minimum dose of PIP in this age group to 59 mg/kg would halve the risk of treatment failure and ensure cure of at least 95% of young children (Figure 5).

Pharmacokinetic studies highlight that the absorption, elimination, and protein binding of several antimalarials vary with both age and weight [15],[48]–[50]. The blood concentrations of long acting antimalarials 7 days after commencing antimalarial treatment correlate with the area under the curve and time during which blood concentrations exceed the minimum inhibitory concentration of the parasite; these have been shown to provide a useful predictor of treatment failure [15],[45]. Blood concentrations of PIP on day 7 are consistently lower in children than adults, a consequence of a smaller volume of distribution, higher clearance, and shorter elimination half-life [15],[45],[51], and this correlates with an increased risk of clinical failure [45]. The lack of a paediatric formulation compounds this further, since dosing by whole or half tablets and in young children can result in a high proportion of children at the extremes of weight or age bands receiving either too high or too low doses [45].

Our pooled analysis highlights that each mg/kg unit decrease in DHA dose is associated with a 20% increased risk of remaining parasitemic on day 3. Furthermore administration of a DHA dose below the WHO recommended 6 mg/kg was associated with a 1.6-fold greater risk of having microscopically detectable gametocytes on day 7. Several previous studies have shown DP to be associated with greater gametocyte carriage compared to other ACTs [12],[22],[32], and a higher risk of parasite transmission to mosquitoes compared to artemether-lumefantrine [31]. This may reflect the lower total dose of DHA in DP compared to that of artemether dose in artemether-lumefantrine [11],[52].

Our analysis suggests that increasing the dose of both DHA and PIP is likely to improve the cure rate of DP in young children and increase gametocyte clearance, both important considerations in reducing parasite transmission particularly in areas where malaria elimination is being pursued. Young children from 1 up to 5 years currently are at the highest risk of being dosed below the WHO recommended minimum of 48 mg/kg, and the lowest PIP exposure for any given mg/kg dose, when compared with older children and adults [15]. However any revision of dosage recommendations must also consider available pharmacokinetic data and careful appraisal of the tolerability and safety of PIP in the key target populations. In this context concerns have been raised by a limited number of studies which have previously documented a dose relationship between PIP and both gastrointestinal and electrocardiographic adverse effects [14]. Analysis of adverse events can be difficult to address retrospectively since these parameters vary in their definition and are often subjective. Data on the more objective measures of gastrointestinal tolerability were available in ten studies included in our pooled analysis. Reassuringly there was no evidence that increasing the dose of PIP had any significant impact on acute vomiting of medication, history of vomiting or diarrhoea.

Our clinical findings and those of complementary pharmacological studies suggest that it is inappropriate to recommend a single target dose of PIP across all age or weight ranges. Retrospective studies of parasites from areas where sulfadoxine-pyrimethamine (SP) was adopted as first line treatment for malaria reveal a cautionary tale. Early evidence of incipient resistance was present long before SP treatment failure became manifest at high levels. It is highly likely that the sub-optimal dosing in young children hastened the demise of SP as a useful antimalarial [7],[48]. The deployment of DP should not relegate this important ACT to a similar history.

Our study has a number of limitations. Although the clinical data used in the analysis constitute almost 70% of the relevant published literature on this treatment regimen, eight studies (2,100 patients) and 170 patients from targeted studies were not available. No specific reasons were given by the investigators for being unable to join the study group, but the majority of these were from Asia. Comparison of the more complete dataset from Africa showed no regional differences in our analysis suggesting that a systematic attrition bias was unlikely. Another limitation of the study is that only in 23% (1,669/7,072) of patients, could drug doses be calculated from the actual number of tablets administered, the total dose in the remainder being extrapolated from the number of tablets predicted to have been administered according to age and weight criteria defined in the study protocol, assuming complete adherence by the attending clinical staff. Although errors in drug administration may have occurred these were generally identified and the patients censored from analysis. Reassuringly, a sensitivity analysis of a subgroup of patients in whom the exact number of tablets was recorded generated identical parameters to the models of the complete dataset. Another limitation arose from DP being a fixed dose combination, making it impossible to determine whether the observed treatment effects were attributable to the dose of PIP, DHA, or a combination of both. The initial reduction in parasite biomass is widely regarded as being determined by the artemisinin derivative because of its significantly higher potency and faster action, whereas the parasite biomass remaining after 3 days of artemisinin exposure is dependent on its elimination by the intrinsically less active partner drug with longer half-life. This is not always the case since several longer acting partner drugs have been shown to contribute to the initial parasite clearance following ACTs [53]. Disaggregating these effects will be important if this combination were to be reformulated with different DHA:PIP ratios.

Our analysis highlights the power of pooled analyses from diverse clinical settings to assess geospatial and temporal trends in antimalarial efficacy. These observations provide critical information for national and international policymakers. Our study confirms that DP is an important addition to the malaria pharmacopoeia; however although its overall efficacy is high, young children are vulnerable to receiving an inadequate dose of PIP and this is associated with an increased risk of recrudescence, prolonged parasite positivity, and greater gametocyte carriage. Together these constitute a potential threat to the useful therapeutic life of one of our most valuable ACTs and suggest that further dose optimisation studies in young children are warranted, including detailed pharmacokinetic evaluation and safety monitoring to ensure the tolerability of any proposed increase in dose. Preservation of the longest possible therapeutic life for our antimalarial armamentarium must be one of the highest priorities for achieving the global elimination of malaria.

## Supporting Information

Text S1 **References of all DP clinical trials, their study designs, and dosing schedules.**
(XLSX)

Text S2 **Maps showing locations of published DP clinical efficacy studies and the studies included in the pooled analysis.**
(PDF)

Text S3 **WWARN clinical data and management statistical analytical plan.**
(PDF)

Text S4 **Transmission classification.**
(XLSX)

Text S5 **WWARN DP data and management statistical analytical plan.**
(PDF)

Text S6 **Authors and contributions.**
(XLSX)

## Abbreviations

ACT: artemisinin combination therapy
AHR: adjusted hazard ratio
AOR: adjusted odds ratio
DHA: dihydroartemisinin
DP: dihydroartemisinin-piperaquine
HR: hazards ratio
IQR: interquartile range
OR: odds ratio
PAR: population attributable risk
PIP: piperaquine
WWARN: WorldWide Antimalarial Resistance Network
