# Correction: The Effect of Dosing Regimens on the Antimalarial Efficacy of Dihydroartemisinin-Piperaquine: A Pooled Analysis of Individual Patient Data

**DOI:** 10.1371/annotation/3db421e4-3e27-4442-8092-2ad1b778f371

**Published:** 2013-12-24

**Authors:** The WorldWide Antimalarial Resistance Network (WWARN) DP Study Group

There was an error in the affiliation for Steffen Borrmann in the acknowledgements. Dr Borrmann’s affiliation is currently listed as Heidelberg University, but should read "Department of Microbiology, Magdeburg University School of Medicine, Germany".

