# Supplementary material for: The Effect of Dosing Regimens on the Antimalarial Efficacy of Dihydroartemisinin-Piperaquine: A Pooled Analysis of Individual Patient Data
Source: PLoS Med. 2013 Dec 3;10(12):e1001564. doi: 10.1371/journal.pmed.1001564 (PMC3848996; doi:10.1371/journal.pmed.1001564)
Supplement: Text S2 — Maps showing locations of published DP clinical efficacy studies and the studies included in the pooled analysis. (PDF) [file pmed.1001564.s002.pdf]

Study sites for clinical trials with Dihydroartemesinin-Piperaquine

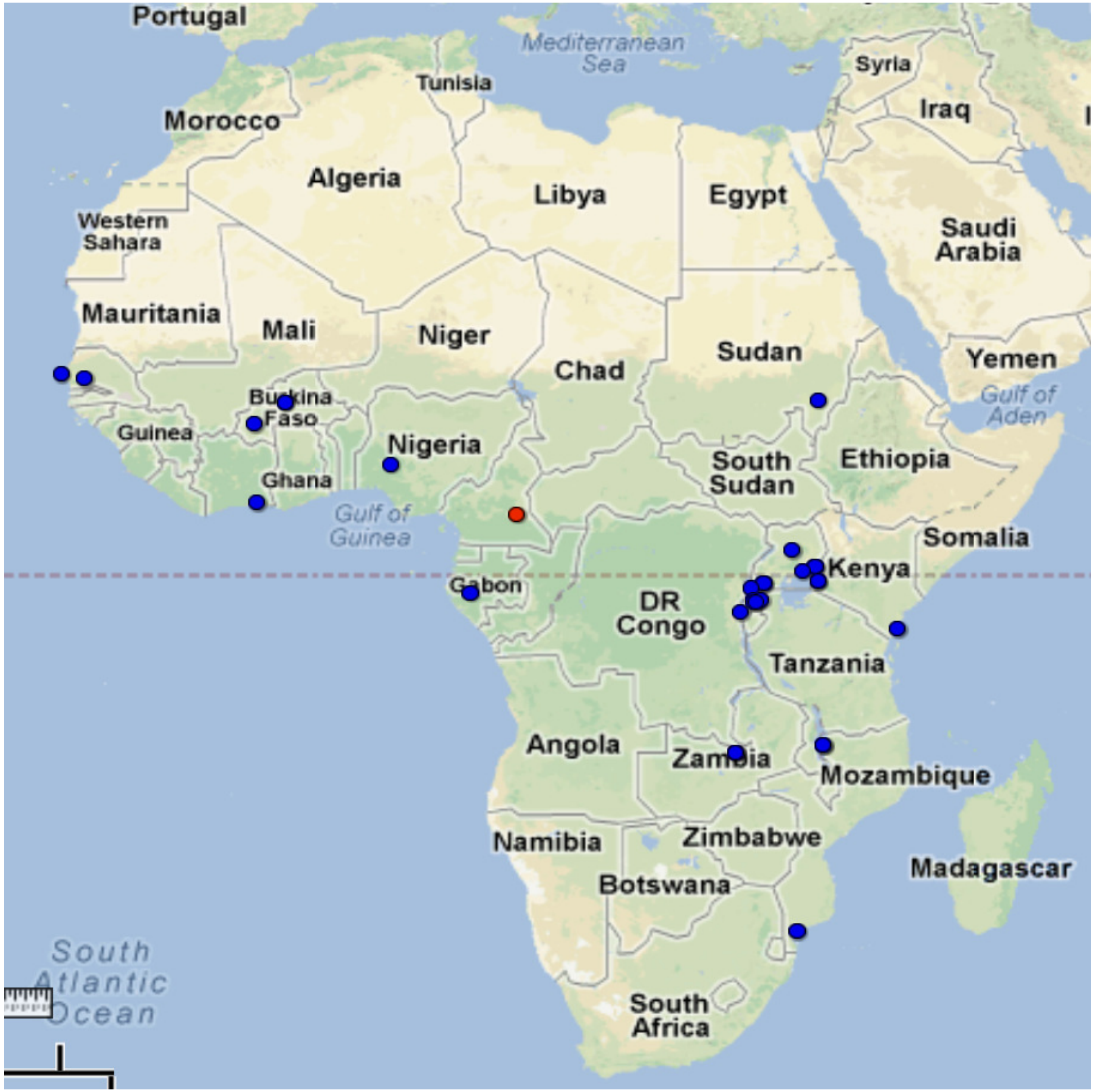

Study sites for clinical trials with Dihydroartemesinin-Piperaquine

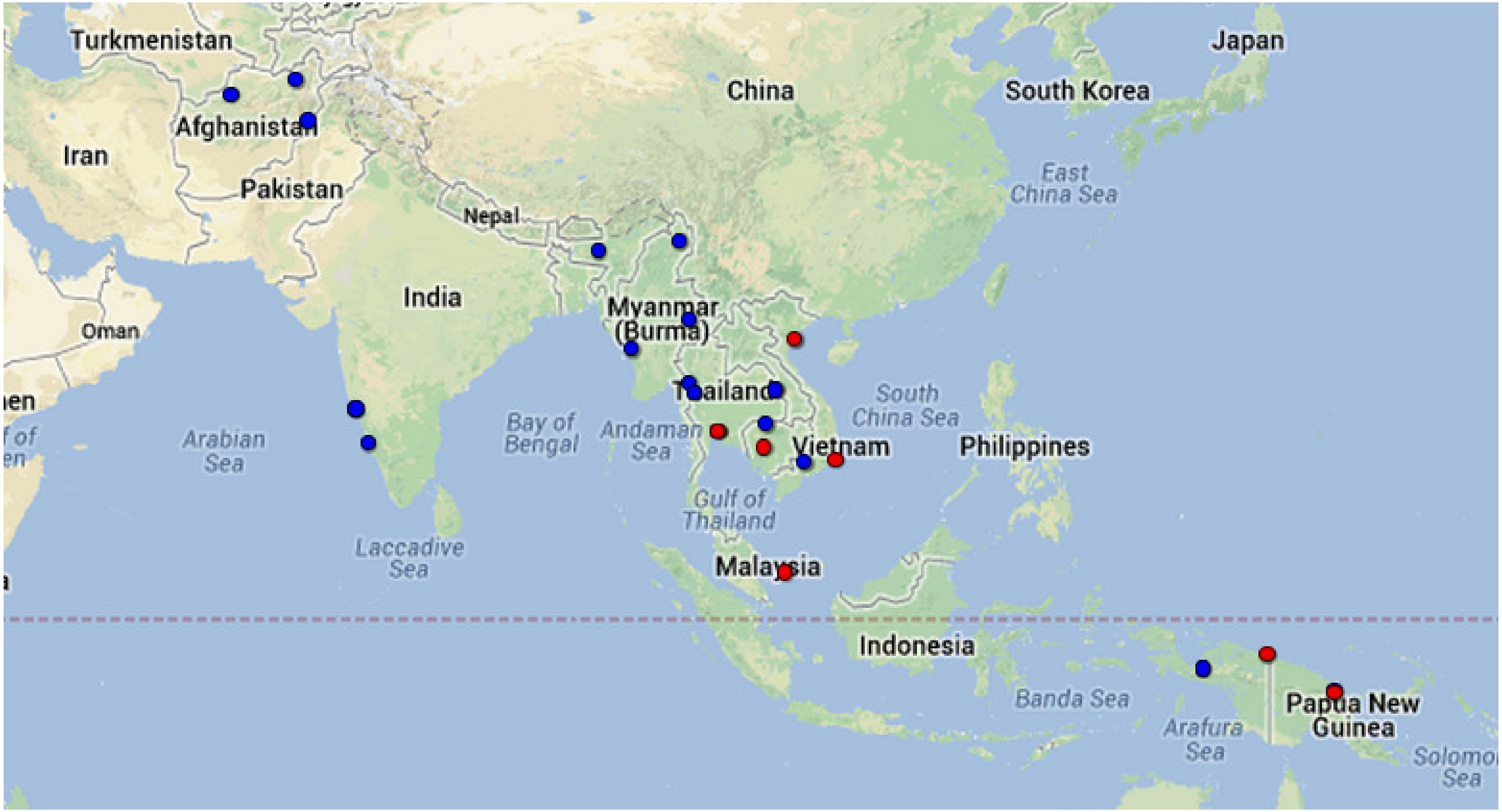

Blue icons represent sites included in the analysis.
Red icons represent sites from publications not included in the analysis

Study sites for clinical trials with Dihydroartemesinin-Piperaquine

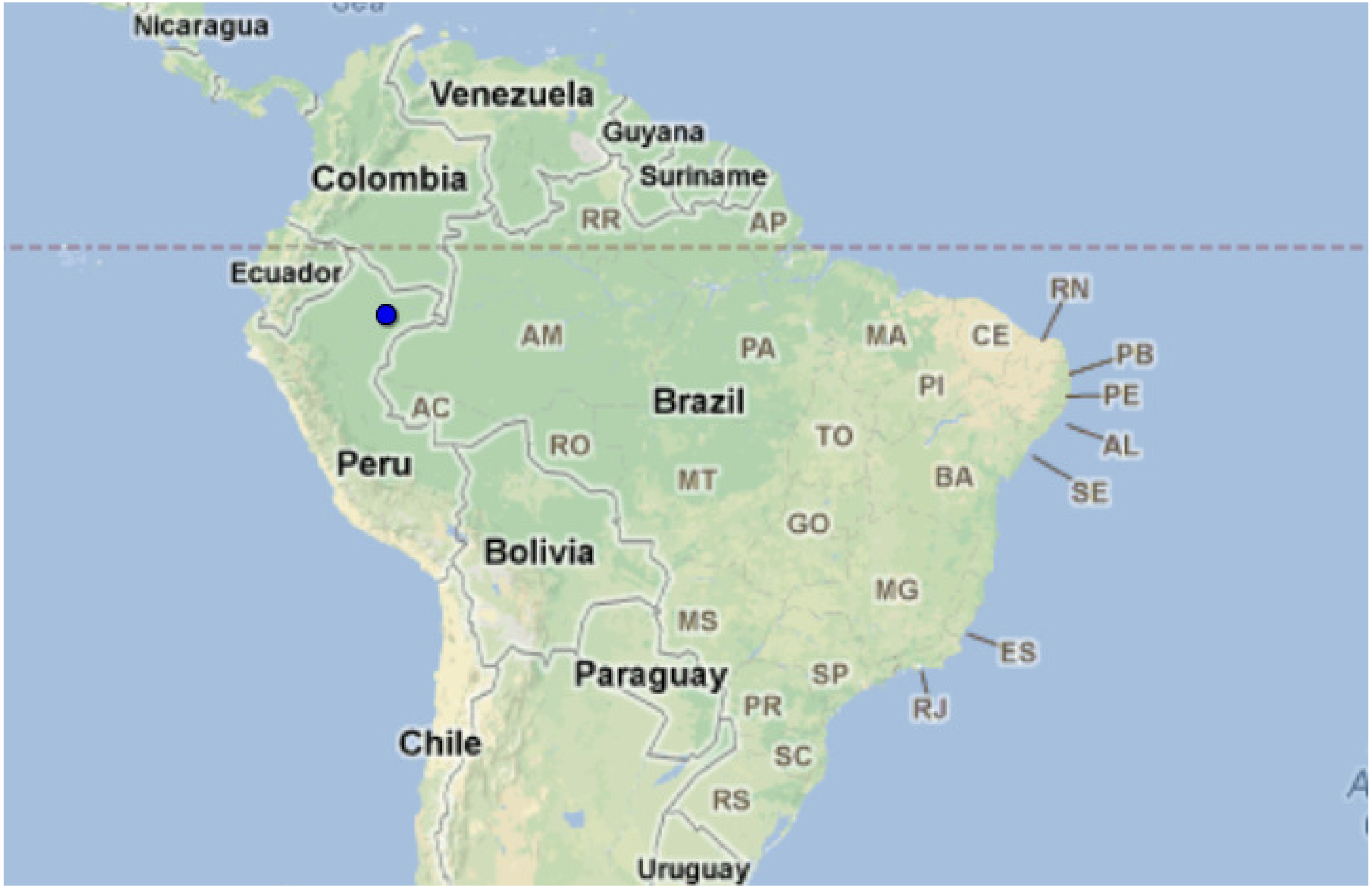

Blue icons represent sites included in the analysis.
Red icons represent sites from publications not included in the analysis
