# Supplementary material for: The Effect of Dosing Regimens on the Antimalarial Efficacy of Dihydroartemisinin-Piperaquine: A Pooled Analysis of Individual Patient Data
Source: PLoS Med. 2013 Dec 3;10(12):e1001564. doi: 10.1371/journal.pmed.1001564 (PMC3848996; doi:10.1371/journal.pmed.1001564)
Supplement: Text S3 — WWARN clinical data and management statistical analytical plan. (PDF) [file pmed.1001564.s003.pdf]

Clinical Module: Data Management and Statistical Analysis Plan
Version 1.2

Clinical Module
WorldWide Antimalarial Resistance Network (WWARN)

Suggested citation: Clinical Module, WWARN, 2012. Data Management and Statistical Analysis Plan.

Version History

Version number	Revision(s) & reason for amendment	Release date
**v1.0**		**4/18/2011**
**v1.1**	Minor changes to the use of PCR results in adjusted analysis (section 7.2) and analysis population (section 8). Added links to Clinical Reports and Patient Book which are now available online.	**8/18/2011**
**V1.2**	Section 7.1 altered to explain definition of efficacy endpoints when more than one parasitemia and temperature measurement per day is recorded. Added link to Parasite Clearance Estimator (section 9.2) which is now available online. Section 11.2 edited to reflect new WWARN process for approval of results. All edits are double underlined.	**5/25/2012**

For more information, contact:
clinical@wwarn.org

WorldWide Antimalarial Resistance Network (WWARN)
www.wwarn.org

Contents

1. Scope 4
2. Introduction 4
2.1 Generating evidence of antimalarial drug efficacy 4
2.2 WWARN's process of data collation 5
3. Dataset submission process 5
3.1 Variables 5
3.2 Online submission system 6
4. Data extraction to a standardised format 7
4.1 WWARN clinical dictionary 7
4.2 Database structure 7
4.3 Data transformation and extraction 8
5. Data cleaning 10
5.1 Unexpected results 10
5.2 Missing values on essential variables 10
6. Deviation definitions 11
6.1 Enrolment deviations 12
6.2 Lost to follow up 12
7. Definitions of efficacy endpoints 13
7.1 WHO definition of efficacy outcome 13
7.2 Plasmodium *falciparum* treatment failure 13
7.3 Plasmodium *vivax* treatment failure 14
7.5 Survival analysis 15
7.6 Parasite clearance measures 16
7.7 Other efficacy parameters 16
8. Analysis population 16
9. Methods of efficacy analysis 17
9.1 Efficacy 17
9.2 Parasite clearance 17
10. Clinical reports and Patient Book 17
10.1 Clinical Data Management and Analysis Report 17
10.2 Justification of data correction 18
10.3 Study report 19
10.3 Patient Book 19
11. WWARN Explorer 19
11.2 Approval of results 19
11.3 WWARN Explorer outputs 19
12. DMSAP versioning 20
12.1 A set of variables (the clinical dictionary) 20
12.2 Management of missing values 20
12.3 Outputs 20
12.4 Integration of cross modular information into a common platform 20
12.5 Pooled analysis of individual patient data 20
13. Conclusion 21
14. References 21
Annex A: Clinical dictionary 22
Annex B: Recoding of string variables 23
Annex C: Example of patient book 24

1. Scope

A key aim of the Worldwide Antimalarial Resistance Network (WWARN) is to monitor geospatial and temporal trends in antimalarial drug resistance. To achieve this, clinical efficacy data need to be collated at an individual patient level so that standardized methodologies and definitions can be applied.

The purpose of the **Clinical Module Data Management and Statistical Analytical Plan** (DMSAP) is to present a clear and transparent methodology by which WWARN handles and analyses clinical data. Documenting the entire process by which data is uploaded, transformed, analysed and presented ensures reproducibility. It also provides a framework for discussing and developing such methodology. WWARN hopes that this process will facilitate best practice, timely data publication and allow geospatial and temporal comparison of clinical data.

2. Introduction

Clinical drug efficacy is a key parameter to formulate malaria treatment guidelines. The WWARN Clinical Module aims to enhance the amount of clinical data available, increase the scientific capacity in endemic countries and provide intelligence on resistance or susceptibility to various antimalarials.

The Clinical Module is working towards these goals as follows:

- Facilitating the inclusion in a **Data Repository** of results from clinical drug efficacy studies carried out by research groups, Non-Governmental Organisations (NGOs) or National Malaria Control Programmes (NMCPs)
- Creating **standardised processes** to facilitate the collection of diverse datasets from studies taking place around the world
- **Optimising analytical tools** to accommodate this diversity and increase comparability of results between heterogeneous studies
- Developing standardised methods and systems to improve the **quality** of source data

2.1 Generating evidence of antimalarial drug efficacy

The World Health Organization (WHO) publishes regularly updated, international guidelines for determining therapeutic efficacy of antimalarial drugs. The most recent international guidelines for monitoring and surveillance of antimalarial drug were published in 2009 (see References). Most research groups, NGOs and NMCPs adhere to these guidelines; however study design modifications (ranging from minor to major) are often adopted by investigators to accommodate specific research questions, characteristics of the study site or simple logistical constraints. To confound matters further, the analysis of clinical efficacy data may vary depending upon whether the data come from a stand-alone drug study or a comparative drug trial. Such variations in the design, methodology, analysis and presentation of clinical drug trials can lead to substantial bias in the derived estimates of drug efficacy as illustrated by Verret *et al* (2009). The interpretation of aggregated data from published results of clinical drug studies is therefore fraught with confounding factors and these aggregated data cannot be used reliably for assessing geographical or temporal trends. Over the last two decades there have been several revisions of guidelines and statistical approaches for the analysis of clinical trials. The collation of raw data from clinical trials will help to ensure that any future methodological changes can be accommodated and presented with minimal effort.

2.2 WWARN's process of data collation

WWARN aims to facilitate two processes: to give researchers the tools to collect, clean and analyse their own data, and to transform clinical data from a diverse range of studies into a common format that can be derived from almost all database structures, so that data from different studies can be pooled and analysed collectively in a standardised manner.

To achieve these goals, a series of steps are followed.

I. Upload individual anonymised patient data from a clinical study
II. Transform uploaded data to a common format
III. Check and identify unexpected data points
IV. Data revision - either by the data submitter, or automatically using a set of rules to autocorrect unexpected data points and neutralise their impact on the analysis
V. Data analysis, applying uniform analytical methodology and reporting to provide consistent estimates of efficacy. These are provided as an automated report to the data submitter. If the data submitter agrees, the data may be presented on the WWARN Explorer, an interactive, online tool which allows users to perform custom queries of more than a hundred studies and visualise the results using dynamic mapping.

WWARN notes that other researchers may take different approaches to data management, particularly with regard to defining and managing protocol deviations. It is very important to stress that it is inevitable that the WWARN-derived efficacy estimates will vary to some degree from analyses performed by the data submitter. These differences do not reflect a value judgment as to which analytical approach is correct. The decisions are made only to apply standardised methodologies and minimise bias on geospatial and temporal trends derived from the many studies compiled in the Data Repository.

3. Dataset submission process

The Clinical Module accepts datasets from antimalarial clinical drug studies that have been obtained in accordance with any laws and ethical approvals applicable in the country of origin.

3.1 Variables

The dataset and/or accompanying documents (e.g. protocols, publications) must contain the following information:

I. Unique patient identifier
II. Date of inclusion and days or dates of follow up visits
III. Treatment received
IV. Patient age or date of birth
V. Parasitaemia and species on day 0 and during follow up.
Note: missing values must be distinguishable from zero parasites in blood smear results
VI. Temperature on day 0 and during follow up
VII. Basic information on study site and study design

If these essential variables are not provided or immediately apparent, additional information may be required prior to data processing and output generation. In these situations, WWARN Data Managers will contact the data contributor for clarification.

The Clinical Module also requests additional variables, where available, to assist analysis and output generation including:

I. Baseline patient data
II. Details of treatment administered including source of drugs
III. Clinical assessment including symptoms and examination
IV. Laboratory data
V. Recrudescence/reinfection genotyping result

The full clinical dictionary is available in Annex A.

3.2 Online submission system

Datasets are submitted using the WWARN online submission system. Data contributors must accept the Terms of Submission [available at http://www.wwarn.org/data/usage] during the submission process.

The data submission steps are:

I. **Register a study**: contributors enter their study title and each study is assigned a unique identifier. Contributors can also tick one or more boxes to indicate the type of data submitted (clinical, molecular, *in vitro* and/or pharmacology).
II. **Permissions**: each study may have any number of administrators, assigned by the original data submitter. An administrator can access the study, upload files and edit supplied information.
III. **Files**: contributors are asked to submit data files and supporting documentation including a data dictionary, protocols and publications.
IV. **Publications:** contributors can provide the Pub Med ID, citation or DOI for publications relating to the submitted data.
V. **Acknowledgements**: the names of acknowledged individuals and institutions will appear in the study details displayed in WWARN Explorer.
VI. **Study info**: data submitters are asked to provide information on the study site and study design. They may enter this information themselves or provide protocols and publications which will be used by WWARN Data Managers to extract the relevant data.

4. Data extraction to a standardised format

Submitted data are extracted and transformed into a standardised format, allowing single study analysis, the generation of a study report and, with permission, visualisation of summary study information on WWARN Explorer. The transformed data is stored in a Data Repository for future pooling with other datasets and meta-analyses.

4.1 WWARN clinical dictionary

The WWARN clinical dictionary contains clinical variables required for generation of efficacy outcomes. A copy of the clinical dictionary is attached in Annex A.

4.2 Database structure

The WWARN clinical dictionary is arranged in six tables linked by a unique patient identifier and date of inclusion. The tables, with examples of component variables, are:

I. **Subject**, e.g. patient age, weight
II. **Treatment**, e.g. investigational product, dose
III. **Laboratory**, e.g. haemoglobin, platelet count
IV. **Parasitology**, e.g. species, parasitaemia, gametocytaemia
V. **Clinical**, e.g. body temperature, splenomegaly
VI. **Molecular**, e.g. genotype result

Submitted data are stored in the Data Repository within these six tables. Data within the Clinical Module can be linked to associated molecular, pharmacology and *in vitro* data using the study and patient identifiers.

Each line in a table is one event with patient identifier, event date or date of inclusion. The subject table shows one line per patient. The treatment table may show one line per patient for a single dose. If dose information on different dates is available there will be more than one line per patient. The parasitology table (below) has a line for each blood smear result of a patient.

Study identifier	patient identifier	date of event	gametocytaemia	parasitaemia (falciparum)
sid	psid	date	gmmicl	pfmicl
GHYDX	3014	06/09/2006	480	4400
GHYDX	3014	08/09/2006	840	0
GHYDX	3014	09/09/2006	512	0
GHYDX	3014	13/09/2006	80	0
GHYDX	3014	20/09/2006	0	0
GHYDX	3014	27/09/2006	0	0
GHYDX	3014	04/10/2006	0	0
GHYDX	3014	11/10/2006	0	0
GHYDX	3014	18/10/2006	0	0
GHYDX	3015	06/09/2006	0	151200
GHYDX	3015	08/09/2006	0	0
GHYDX	3015	09/09/2006	0	0
GHYDX	3015	13/09/2006	0	0
GHYDX	3015	20/09/2006	0	0
GHYDX	3015	27/09/2006	0	0

Figure 1: Example of Clinical Module Parasitology data table

4.3 Data transformation and extraction

Source data may be presented as flat files, with one line per patient, or multiple relational databases. The extraction process transforms all source datasets into a common standard format. Variables from the source dataset, equivalent to variables in the WWARN clinical dictionary, are extracted and imported into one of the six tables. All string variables are systematically recoded as shown in Annex B. An audit trail records and saves the complete data extraction and transformation process. Once transformed, the submitter or their designees may download the derived dataset in the standard WWARN format that is amenable to other datasets submitted to WWARN, for offline analysis. Some important considerations in the data transformation process are highlighted below.

4.3.1 Date of inclusion

If the date of inclusion or visit dates per patient are not specified, i.e. there are no dates present in the dataset, a theoretical date of inclusion - January 1 [study year] is used for each patient. This date is used to tag the patient to a specific year and to derive the subsequent dates of follow up.

4.3.2 Splenomegaly and hepatomegaly

Splenomegaly and/or hepatomegaly noted in centimetres in the source dataset are converted to a binary (yes/no) variable. For example, if the source dataset notes splenomegaly >0 cm, this is converted to the variable splenomegaly=yes. The rationale for this approach is that the size of organomegaly is often poorly quantified by clinical examination. It is the presence or absence of organomegaly, rather than its degree, that appears to have greater clinical relevance.

4.3.3 Previous antimalarial intake

Prior antimalarial intake is an important risk factor for selection of drug resistance and subsequent recrudescence although recording is vulnerable to recall bias. Study designs have used a wide variety of measures and methods (e.g. urine confirmation of drug levels) to quantify this parameter.

The corresponding WWARN variable is "A*ntimalarial intake in the last 28 days?"* A source variable of previous antimalarial intake must be contained within this time frame. Therefore, a source variable "*Antimalarial intake in the last 40 days?"* would not be equivalent. A "yes" in a source variable of "*Antimalarial intake in the last 7 days"* would translate to "yes" for the WWARN variable "*Antimalarial intake in the last 28 days"*. If "*Antimalarial intake in the last 7 days"* is "no" then the WWARN variable will be unknown. The table below demonstrates transformations of these variables.

**Source variable**	**Source response**	**WWARN variable**	**WWARN recoding**
Antimalarial in the last 7 days?	Yes	*Antimalarial in the last 28 days?*	Yes
Antimalarial in the last 7 days?	No	*Antimalarial in the last 28 days?*	Unknown
Antimalarial in the last 28 days?	Yes	*Antimalarial in the last 28 days?*	Yes
Antimalarial in the last 28 days?	No	*Antimalarial in the last 28 days?*	No
Antimalarial in the last 40 days?	Yes	*Antimalarial in the last 28 days?*	Unknown
Antimalarial in the last 40 days?	No	*Antimalarial in the last 28 days?*	No

If the source data has many variables capturing information about previous antimalarial intake, e.g. "C*hloroquine in the last 28 days?"* and *"Mefloquine in the last 28 days?"*, then these variables will be combined into the one WWARN variable "*Antimalarial intake in the last 28 days?*" – see the following Table.

**Source variable name**	**Source response**	**Source variable name**	**Source response**	**WWARN variable**	**WWARN recoding**
Chloroquine in the last 28 days?	Yes	*Mefloquine in the last 28 days?*	Yes	*Antimalarial in the last 28 days?*	Yes
Chloroquine in the last 28 days?	Yes	*Mefloquine in the last 28 days?*	No	*Antimalarial in the last 28 days?*	Yes
Chloroquine in the last 28 days?	No	*Mefloquine in the last 28 days*	Yes	*Antimalarial in the last 28 days?*	Yes
Chloroquine in the last 28 days?	No	*Mefloquine in the last 28 days?*	No	*Antimalarial in the last 28 days?*	No

4.3.4 History of fever

Similar diversity exists with documenting prior history of fever. WWARN has defined a variable "*Fever in the last 24 hours?"* and source variables of fever history must be within this time frame to be deemed equivalent.

4.3.5 Drug administration

Drug dose is recorded in multiple WWARN variables to ensure the importation system can capture the variety of ways this information is presented in source datasets. WWARN dosage variables include; total mg per day, mg per kg, tablets per day, tablets per dose, total number of doses, doses per day. Variables for drug vomiting within one hour of administration (yes/no) and re-dosing (yes/no) for each event of drug administration are included in the WWARN clinical dictionary.

4.3.6 Species and parasitaemia

Although WHO recommends that only single species infections are included in drug efficacy studies, mixed infections are extremely common outside of Africa, and are often included in clinical trials. Quantification of each sub-infection by microscopy is challenging since species differentiation is extremely difficult and often unreliable at young ring stage. WWARN captures information on the asexual stages (species of infection and asexual parasitaemia) and sexual stages (species of infection and gametocyte count). For mixed infections the total asexual or sexual parasitaemia is collected.

Parasitaemia can be quantified by thick or thin blood film examination, or counting per 200, 500 or even 1000 white blood cells (WBC) or 1000 red blood cells (RBC). Raw parasite data are converted into parasite density per µl of blood according to the following formulae:

- Parasitaemia = (Parasite count per N WBC/N) * WBC
  If WBC is missing then a count of 8,000 will be assumed.

Or

- Parasitaemia = Parasite count per 1000 RBC * 125.6 * Haematocrit
  If haematocrit is missing then a value of 33% is assumed.

5. Data cleaning

Data are checked for **inconsistencies**, **unexpected values** and **missing values**. If identified, these values are fed back to the data contributor, along with the patient identifier, for possible correction from source documents. Resubmitted, corrected, and/or transformed missing values will be used to update the Data Repository. If corrections cannot be made, the inconsistencies and unexpected results are transformed to missing values. In order to transform all data sets into a consistent format for potential combined analysis, variables are constrained within limits set by the analysis program as described below.

5.1 Unexpected results

5.1.1 *Single variable*

The following single variable data checks are conducted on all studies. In general these extreme values are deemed incompatible with the range of values observed in malaria clinical trials.

I. Temperature < 34°C
II. Temperature > 42°C
III. Age > 90 years old
IV. Haemoglobin > 25 g/dL
V. Haematocrit > 50%
VI. Parasitaemia > 500,000
VII. Weight > 120 kg

5.1.2 Combined variables

In addition to these single variable data checks, the following combined variable data checks are conducted on all studies.

I. Weight > 100 kg for patients aged 5–14 years
II. Weight > 50 kg for patients under 5 years
III. Weight < 10 kg for patients older than 15 years
IV. Weight < 5 kg for patients aged 5–15 years
V. Weight < 1 kg for patients under 5 years
VI. Patient with recurrent parasitaemia but missing PCR result for studies containing genotyping data
VII. Patient with no recurrent parasitaemia but PCR result indicates recrudescence or reinfection

In the case of patient age and weight discordance, weight will be converted to a missing value and the age value will be accepted as correct.

5.2 Missing values on essential variables

The following variables are checked for missing values and a query generated for the submitter.

I. Treatment
II. Gender
III. Enrolment date

6. Deviation definitions

A protocol deviation is an unintentional action or process, which may affect the integrity of data and antimalarial efficacy results. There is often confusion between the terms “deviation” and “violation”. In this document, these two terms have the same meaning, the emphasis being on the deviations effect on the efficacy outcome (censoring, failure or no effect). Examples of deviations include:

I. Inclusion/exclusion criteria not fulfilled
II. Administration of medication with antimalarial activity during follow up
III. Missing blood smears
IV. Patient failing to comply with study requirements such as follow-up visits

Given the diversity of datasets and studies processed by WWARN, the use of source deviations from each study protocol may lead to bias in efficacy measurement. Therefore, differences in derived efficacy estimates may be caused by different study deviations rather than due to the emergence of resistance *per se*. As an example, some study protocols may document use of a concomitant medication with antimalarial activity during follow-up and censor the data; other studies may not actively capture this information. In addition, studies may be designed for different purposes, e.g. a comparative analysis of two treatments or resistance monitoring of a single drug regimen. In the former case, a protocol deviation such as early intolerability from vomiting might be regarded as a treatment failure requiring rescue therapy. For a standalone resistance study, these cases would represent censorship on the day of rescue rather than treatment failure.

To accommodate these scenarios WWARN has developed a standardised set of deviations that are applied to all studies. The application of a standard approach to manage deviations will help to minimise the bias of original study design when comparing between different studies. However it will inevitably result in estimates of antimalarial efficacy that differ from the original protocol analysis and prior publications. Usually these differences will be small, but in some circumstances the difference may be noticeable (Verret *et al*, 2009). It is extremely important to note that any differences in derived results are likely to reflect variance in analytical methodologies. However, standardised methodology is critical for pooled analysis and is the rational of this approach.

Protocol deviations can be classified into different categories depending upon their impact on patient health or impact on efficacy results. WWARN focuses on common deviations which primarily affect efficacy results. Patients are censored on the day the deviation occurred.

WWARN study deviations are presented in two groups: at enrolment and during follow-up. There are other important deviations that are currently not captured systematically in the WWARN Data Repository, for example, “*Use of other antimalarials during follow up”*, “*Patient withdrawal of consent”* or “*Treatment protocol deviations”.* The diverse definitions of these deviations in the source datasets does not allow them to be accurately captured in a manner that is standardised and comparable across studies. This limitation to the WWARN methodology may be addressed in future version of the WWARN Clinical DMSAP.

6.1 Enrolment deviations

Deviation	Effect
Hb < 5 g/dL on day 0	Censored on day 0
Ht < 15% on day 0	Censored on day 0
"*Severe anaemia*" variable labelled as "*Yes*" on day 0	Censored on day 0
Hyperparasitaemia, defined as parasite density > 250,000/µL	Censored on day 0
No parasitemia on day 0<br>Note: parasitemia > 500,000 is converted to a missing value	Censored on day 0

6.2 Lost to follow up

Deviation	Effect
More than 18 days without blood smear results	Lost to follow up on day of last visit
No blood smear results within [D25;D31] in a D28 study	Lost to follow up on day of last visit for day 28 efficacy outcomes
No blood smear results within [D39;D45] in a D42 study	Lost to follow up on day of last visit for day 42 efficacy outcomes
No blood smear results within [D60;D66] in a D63 study	Lost to follow up on day of last visit for day 63 efficacy outcomes

7. Definitions of efficacy endpoints

7.1 WHO definition of efficacy outcome

WWARN uses the WHO definitions of efficacy outcome as defined below:

Early treatment failure (ETF)

- Danger signs or severe malaria on day 1, 2 or 3, in the presence of parasitaemia
- Parasitaemia on day 2 higher than on day 0, irrespective of axillary temperature
- Parasitaemia on day 3 with axillary temperature ≥ 37.5°C
- Parasitaemia on day 3 ≥ 25% of count on day 0

In the case of multiple daily parasitaemia or temperature measurements, any temperature or parasitaemia on day 1, 2 or 3 that defines an early treatment failure will be prioritised over other measurements occurring on the same day.

Late clinical failure (LCF)

- Danger signs or severe malaria in the presence of parasitaemia on any day between day 4 and last day of follow up in patients who did not previously meet any of the criteria of early treatment failure
- Presence of parasitaemia on any day between day 4 and last day of follow up with axillary temperature ≥ 37.5°C in patients who did not previously meet any of the criteria of early treatment failure

Late parasitological failure (LPF)

- Presence of parasitaemia on any day between day 7 and last day of follow up with axillary temperature < 37.5°C in patients who did not previously meet any of the criteria of early treatment failure or late clinical failure

Adequate clinical and parasitological response (ACPR)

- Absence of parasitaemia on the last day of follow up , irrespective of axillary temperature, in patients who did not previously meet any of the criteria

If a follow-up visit occurs 3 days before or 3 days after the scheduled visit e.g. a day 28 visit occurs between day 25 and day 31, the outcome is classified on the day of scheduled visit (day 28) for the purposes of reporting efficacy outcomes.

7.2 Plasmodium *falciparum* treatment failure

In the **PCR adjusted analysis of *P. falciparum* treatment failure**, patients enrolled with *P. falciparum* infection will be included in the analysis with the following considered as treatment failures:

- Early treatment failures
- *P. falciparum* parasitaemia between 4 and 7 days after treatment unless PCR results indicate reinfection
- Late treatment failure (late clinical failure or late parasitological failure) after day 7 with *P. falciparum* (alone or mixed) with PCR-confirmation of recrudescence

Patients with recurrent parasitemia after day 7 and without PCR confirmed recrudence or reinfection will be censored on the day of recurrent parasitemia.

In the **unadjusted analysis of *P. falciparum* treatment failure**, patients enrolled with *P. falciparum* infection will be included in the analysis with the following considered as treatment failures:

- Early treatment failures
- Any recurrent *P. falciparum* parasitaemia from day 4 until the end of the follow-up period

Patients presenting as a study deviation (see Section 6.1) will be censored on the day it occurred. If a patient has multiple deviations at different times, s/he will be censored at the time of the first deviation.

7.3 Plasmodium *vivax* treatment failure

The use of PCR genotyping to identify recrudescent *P. vivax* infections is confounded by the presence of both reinfections and relapses. As yet there are no agreed methods of PCR correction and hence only the PCR unadjusted risk of *P. vivax* treatment failure will be presented.

In the **unadjusted analysis of *P. vivax* treatment failure** patients enrolled with *P. vivax* infection will be included in the analysis with the following considered as treatment failures:

- Early treatment failures
- Any recurrent *P. vivax* parasitaemia after day 4 until the end of the follow-up period

Patients presenting as a study deviation (see Section 6.1) will be censored on the day they occurred. If a patient has multiple deviations at different times, s/he will be censored at the time of the first deviation.

7.4 Inclusion of mixed species infections

Mixed species infections may influence the derived estimates of treatment efficacy and for this reason the WHO guidelines recommend that only single species infections are included in drug efficacy studies. However, in practice, the effect of mixed infections is usually minor. Outside Africa, mixed infections are extremely common, and may represent an important population group in which to define antimalarial efficacy. WWARN does not seek to interfere with the study design proposed by the investigator. Instead, results will be generated for the complete dataset. However it should be noted that subgroup analyses (e.g. analysis of patients presenting only with pure *P. falciparum*), can be easily generated. For example, in the WWARN Explorer, study results can be generated after filtering by the species of infection. Where mixed infections have been included in a study, Kaplan-Meier efficacy estimates will be derived from patient data according to the following tables where 0 = censored on the day of recurrent parasitemia and 1 = failure, “, assuming that all recurrent parasitemia before day 7 will be considered recrudecense unless PCR results indicate otherwise.

P.falciparum* efficacy estimate*:

Day 0 species	Day of failure species	PCR	PfAdj	PfUndj
Pf	Pf	Recrudescence	1	1
Pf	Pf	Reinfection	0	1
Pf	Pf	No PCR	0	1
Pf	Pf + other	Recrudescence	1	1
Pf	Pf + other	Reinfection	0	1
Pf	Pf + other	No PCR	0	1
Pf	Not Pf	No PCR	0	0
Pf + other	Pf	Recrudescence	1	1
Pf + other	Pf	Reinfection	0	1
Pf + other	Pf	No PCR	0	1
Pf + other	Pf + other	Recrudescence	1	1
Pf + other	Pf + other	Reinfection	0	1
Pf + other	Pf + other	No PCR	0	1
Pf + other	Not Pf	No PCR	0	0

P.vivax* efficacy estimate*:

Day 0 species	Day of failure species	Pv
Pv	Pv	1
Pv	Pv + other	1
Pv	Not Pv	0
Pv + other	Pv	1
Pv + other	Pv + other	1
Pv + other	Not Pv	0

7.5 Survival analysis

Patients are followed until they have a treatment failure, are censored due to a study deviation or reach the last day of the study follow up (as defined in the study protocol).

Survival analysis for antimalarial drug resistance uses two parameters - the **status** at the time of censor and the **survival time**.

Status is a dichotomous variable derived from whether the patient fulfils criteria for treatment failure or is censored. The status (0/1) may vary depending on the estimate being derived and the outcome parameter, for example PCR adjusted *P.falciparum*, unadjusted *P. falciparum* or *P.vivax* recurrence.

The survival time, sometimes referred to as censoring time, is the time between the date of inclusion (day 0, day of first drug intake), an efficacy event, for example treatment failure, a censoring point such as a deviation, or the end of follow-up.

7.6 Parasite clearance measures

Analysis of parasite clearance dynamics is a crucial aspect of the therapeutic response, especially relevant to artemisinin resistance monitoring. WWARN is currently developing models and analytical approaches to quantify parasite clearance. Version 1 of the Clinical Study Report will simply report patient parasitaemia on day 2 and day 3, defined as the percentage of patients remaining with parasites on that day. Many studies stop monitoring blood films in the first week once a negative smear has been recorded. Treatment of missing parasitaemia data on either day 2 or day 3 are described in the table below.

**Parasitaemia**				**Effect**
**D0**	**D1**	**D2**	**D3**	
Yes	Yes	No	Missing	Assumed no parasitaemia on day 3
Yes	Yes	Missing	Yes	Assumed parasitaemia on day 2
Yes	No	Missing	Missing	Assumed no parasitaemia on day 2 and 3
Yes	Yes	Missing	No	Will not contribute to proportion of patients with parasitaemia on day 2
Yes	No	Missing	No	Assumed no parasitaemia on day 2
Yes	No	Missing	Yes	Will not contribute to proportion of day 2 with parasitemia
Yes	Yes	Missing	Missing	Will not contribute to proportion of patients with parasitaemia on day 2 or 3

Note: this is only for calculations of the proportions of patients who remain parasitaemic on each day. It is not for the analysis of parasite clearance rate data which may take on a myriad of different analytical structures.

7.7 Other efficacy parameters

Other important parameters of the efficacy response, including fever clearance times, haematological recovery and gametocyte carriage, will be incorporated into future versions of the WWARN Clinical DMSAP.

8. Analysis population

The WWARN analysis population is defined as all study patients not excluded due to deviations on day 0 and whom are included in a treatment arm of 10 patients or more. Treatment arms containing less than 10 patients will be excluded from the analysis. Patients with missing information during follow-up for whatever reason (withdrawal, loss to follow-up, deviation from study protocol) will be included, as data permits, in the analysis described in the following efficacy analysis sections.

9. Methods of efficacy analysis

9.1 Efficacy

Response to treatment (defined in Section 7) is analysed using survival data analysis methods. Kaplan-Meier product limit estimate is used to calculate efficacy or failure rates for each treatment at a given time during follow-up, at least at day 7, 14, 21, 28 and then every seven days until the end of the follow-up period (Kalbfleisch and Prentice, 2002). Confidence intervals for these point estimates are calculated based on the asymptotic variance of the log-log transformed survival function (Kalbfleisch and Prentice, 2002). Status and survival variables defined in Section 7.5 are used for this purpose.

Two analyses are performed - PCR unadjusted and PCR adjusted (if relevant information is available).

In a PCR unadjusted analysis, all recurrences of *P. falciparum* parasitaemia after day 4 until the end of the follow up period and ETFs (or persisting parasitaemias up to Day 7), are treated as treatment failures. All other events during follow-up (*P. vivax* malaria, withdrawal due to adverse event, concomitant medication or any other reason) will result in the patient being censored at the time of this event. Patients lost to follow-up are censored at the time of their last follow-up visit.

The same rules apply to the PCR adjusted analysis except patients who develop a new *P. falciparum* infection after day 6 of the follow-up period will be censored at the time of parasite reappearance. Patients with recurrent parasitaemia and missing PCR information (indeterminate result, missing sample, PCR not done) will also be censored at the time of the parasitaemia recurrence. Kaplan-Meier estimates will be presented in a table and graphically.

9.2 Parasite clearance

WWARN will present both the number (n) and the proportion (%) of patients with a positive parasite count on day 2 and 3 as well as the number (N) of patients evaluated on that day: x% (n/N). WWARN estimates the percentage of remaining positive blood smears during the first 3 days after inclusion. For reporting of patients with missing parasitaemia counts on days 1, 2 or 3, refer to Section 7.6 (Parasite clearance measures).

In studies with frequent (at least every 12 hours) parasite measurements until clearance, the parasite clearance rate and initial parasite clearance lag phase duration are estimated by modelling the log(parasitemia) time profile using the PCT calculator, details of which are available on the WWARN website (http://www.wwarn.org/research/parasite-clearance-estimator).

10. Clinical reports and Patient Book

10.1 Clinical Data Management and Analysis Report

After a dataset has been uploaded and transformed, an automated Data Management and Analysis Report, and a separate Study Report are produced using the standardised data format. Examples are available on the WWARN website (http://www.wwarn.org/research/clinical).

The Data Management Report and Analysis contains:

I. **A basic description of the study** including the total number of participants, by therapeutic group

II. **Information on which source variables** were extracted

III. **Systematic audits**

III.1. **Data consistency**: the numbers of unexpected results (Section 5.1) are tabulated with an annexed, detailed list of cases (patient number, date, day, patient specific data)

III.2. **Study deviations**: the numbers of study deviations (Section 6) are tabulated with an annexed, detailed list of cases (patient number, date, day, patient specific deviation with explanatory data)

III.3. **Data description**: a table presenting the percentage of participants with the following unexpected results:

- More than 18 days between consecutive blood smears
- Recurrent parasitaemia without PCR
- Non PCR-adjusted recurrent parasitaemia
- No blood smear data between day 7 and day 28
- Missing blood smears during follow-up
- Age > 120 years
- Parasitaemia > 500,000/µL
- Unexpected weight for age (see Section 5.1.2)
- No recorded gender
- Temperature > 42 ^{o}C or < 34°C

IV. **Efficacy analysis**: comprising results commonly provided in a report or publication of any antimalarial resistance clinical trial.

IV.1. **Trial profile**: displaying the total number of included patients and study deviations, by study arm. The remaining patients are numerated by study arm: ETF, LTF before day 7, recrudescence, or ACPR. When a patient has two or more deviations, only the first chronological deviation is taken into account.

IV.2. **Baseline characteristics**: from day 0 values

IV.3. **Treatment outcomes**: efficacy outcomes (ACPR, types of treatment failures) and study deviations are tabulated with the corresponding frequencies, by study arm. The results are presented at day 28 and day 42 and the last day of follow up, if different.

IV.4. **Kaplan Meier curves**: the Kaplan Meier survival curves presented graphically and tabulated for PCR-adjusted and unadjusted *P. falciparum* treatment failure or *P. vivax* recurrence.

10.2 Justification of data correction

Baseline characteristics, Kaplan-Meir curves and life tables are all presented using both auto-corrected and the original, non-modified, data. Auto-correction transforms the unexpected results described in Section 5.1 into missing values. In the unchanged mode, all source values sent by the data submitter, which might include an age of 650 years, or a parasitaemia of 10^6/µL, are unchanged and used to produce baseline characteristics and efficacy results.

The submitter will be provided with a spreadsheet for correction of unexpected data points and missing values. Displaying outputs with both auto-corrected and unchanged data allows the data

submitter to readily judge the impact of auto-correction. If auto-correction causes limited or no changes to the efficacy results or baseline characteristics, the submitter may choose not to correct the data with patient files. Although the report will contain both sets of data, the WWARN Data Repository will only utilise the submitter-corrected data and/or auto-corrected data for future study analyses.

10.3 Study report

The Study Report is derived from the Data Management and Analysis Report. It summarises the methodology and results but describes neither the systematic audit nor data inconsistencies. The results are presented only with automatic data correction. The report is provided should the data submitter wish to share their study results with others in the malaria community.

10.3 Patient Book

Data submitters also receive a Patient Book, an example page of which is shown in Annex C. The study history of each participant is provided in a one page summary with the resulting deviations or efficacy endpoints. The Patient Book, which will have as many pages as there are patients in the study, is provided for reference.

11. WWARN Explorer

The WWARN Explorer is an online, open-access web-based tool which allows users to visualise the results using dynamic interactive maps.

11.1 Selected variables for display in WWARN Explorer

Imported variables from each study are used to derive a set of standardised core variables that define efficacy outcomes according to the WHO guidelines for the assessment and monitoring of antimalarial drug efficacy (Price *et al*, 2007). The core variables include: study identifier, patient identifier, age, gender, weight, treatment received, initial *Plasmodium* species of infection, species on last day of follow up, initial parasite density, last day of follow up, derived outcome (see Section 7) and result of any PCR genotyping. The standardised core variables are transferred from the WWARN Data Repository and presented as clinical outputs on the WWARN Explorer.

11.2 Approval of results

Data submitters are able to review results obtained following methodology detailed in the WWARN Clinical DMSAP in the comprehensive Data Management and Analysis Report and may request screenshots of their data, as they will appear on WWARN Explorer. Data submitters who decide not to display their results on the WWARN Explorer should contact WWARN after receiving their Study Reports.

11.3 WWARN Explorer outputs

WWARN explorer will display the following outputs per study:

I. Basic study information (study title, country, year, total enrolment and day 28 success rate)
II. Study profile
III. Graph of patient age ranges and user-selected, age increment table
IV. Table of recurrent parasitaemias showing number of patients affected per *plasmodium* species
V. Table of treatment efficacy at day 28 for each drug using WHO definitions

VI. PCR-adjusted cure-rate chart (where applicable)
VII. PCR-unadjusted cure-rate chart
VIII. Cure-rate table displaying PCR-adjusted cure-rate (%) and unadjusted cure-rates, with 95% confidence intervals on day 7 and day 28, and if applicable, day 42 and 63.

12. DMSAP versioning

12.1 A set of variables (the clinical dictionary)

The variables imported from the data submitter - the clinical dictionary - define the types of analysis that will be possible with the dataset. Version 1 of the Clinical DMSAP focuses on the essential variables necessary to define resistance based on the WHO guidelines. It does not capture additional variables, including:

- Adverse events (also serious adverse events)
- Extensive baseline characteristics that could inform multivariable analysis of risk factors for failure or PK dynamics
- Sub-population specific variables such as gestational age or date of delivery related to pregnancy.

Additional variables may be included in future version of the clinical dictionary and DMSAP.

12.2 Management of missing values

Missing values are omitted from analysis in this version of the Clinical DMSAP. In future versions, multiple imputations or other strategies may be applied to minimise data loss.

12.3 Outputs

Version 1 outputs are described in the sections describing the Clinical Reports (Section 10) and the WWARN Explorer (Section 11). New outputs under consideration for future versions include:

- Evolution of haemoglobin or gametocytes over time, multivariate analysis
- Parasite clearance rate
- Description of adverse events, multivariate analysis with new variables
- Using variables available in the Study Site Questionnaire:
    - Hyperparasitaemic inclusions
    - Treatment failure inclusions
    - Transmission level in the study site

12.4 Integration of cross modular information into a common platform

Some studies have *in vivo*, PK, *in vitro* and/or molecular data documented on individual patients presenting the possibility for "cross-modular" analyses. Work is ongoing so that future versions can report cross modular analysis.

12.5 Pooled analysis of individual patient data

Pooling individual dataset from several clinical trials conducted in different locations and at different times could theoretically yield useful indicators of temporal or spatial trends as the basis for an effective, early warning system of emerging or spreading resistance. However, such meta-analyses will require rigorous statistical approaches. A planned *Pooled Dataset Statistical Analysis Plan* will describe the processes and analytical methods for such pooled analyses although some elements may be included in future versions of the Clinical DMSAP.

13. Conclusion

The Clinical Data Management and Statistical Analytical Plan is an evolving document that aims to be in line with current WHO guidelines on monitoring antimalarial efficacy. However WWARN strives to incorporate data from clinical trials conducted using modified study designs. Furthermore, there are some situations where there is no international consensus on their management. In these cases WWARN has sought advice from a broad group of experts and chosen an approach based on those inputs. There will always remain parts of the methodology which are contentious. WWARN encourages feedback on these issues and will endeavour to incorporate suggestions into future versions or bring major issues into a wider forum for open discussion. Comments should be directed to clinical@wwarn.org.

14. References

Kalbfleisch JD, Prentice RL. The statistical analysis of failure time data. New York: John Wiley & Sons, 2002.

Price RN, Dorsey G, Ashley EA et al. World Antimalarial Resistance Network I: Clinical efficacy of antimalarial drugs. *Malaria Journal* 2007; 6:119.

Stepniewska K, White NJ. Some considerations in the design and interpretation of antimalarial drug trials in complicated falciparum malaria*. Malaria Journal* 2006*;* 5:127.

Verret WJ, Dorsey G, Nosten F, Price RN The effect of varying analytical methods on estimates of anti-malarial clinical efficacy. *Malaria Journal* 2009; 8:77.

World Health Organization 2009. Methods for surveillance of antimalarial drug efficacy. ISBN 978 92 4 159753 1.

Annex A: Clinical dictionary

Baseline patient data

- Unique patient identifier
- Date of inclusion and days or dates of follow up visits
- Patient age or date of birth
- Weight
- Fever history
- History of malaria
- History of antimalarial intake

Clinical assessment including symptoms and examination

- Temperature
- Fever
- Hepatomegaly
- Splenomegaly
- Vomiting
- Signs of severe malaria
- Diarrhoea

Treatment

- Treatment received
- Post treatment vomiting
- Rescue treatment
- Dosage
- Concomitant medication

Laboratory data

- Parasitaemia
- Species
- Gametocytaemia
- Haematology

Recrudescence/reinfection genotyping result

Outcome

Annex B: Recoding of string variables

Gender	Male	M
	Female	F
Species	F	falciparum
	V	vivax
	O	ovale
	M	malariae
	MIX	mixed indistinguishable
PCR result	RI	Reinfection
	RC	Recrudescence
	NA	Not applicable
	NR	No result available
	NPF	Non Pf malaria
	IND	Indeterminate
	O	Other
Clinical outcome	ACPR	ACPR: Complete follow up no failure
	ETFD	ETF with Death
	ETFS	ETF with Severe malaria
	ETFDS	ETF with Danger Signs
	ETFP	ETF with Parasitological criteria
	ETFC	ETF with Clinical criteria
	ETF	ETF not otherwise specified
	LCFD	LCF with Death
	LCFS	LCF with Severe Malaria
	LCFDS	LCF with Danger Malaria
	LCFF	LCF with fever (either measured or subjective)
	LCF	LCF not otherwise specified
	LPF	LPF
	LCFLPF	LPF/LCF Indistinguishable
	AE	Adverse event requiring change in antimalarial therapy prior to completion of full dose of study drug
	PD	Treatment protocol deviation (e.g. incomplete course, wrong dose…)
	D	Death not due to malaria
	LFU	Lost to follow-up
	AMLR	Use of other antimalarial outside of study protocol in the absence of parasitaemia
	CW	Withdrawal of consent by patient prohibiting further follow-up
	IW	Investigator initiated withdrawal from further follow-up (e.g. development of concomitant illness, death unrelated to malaria)
	O	Patient who does not complete follow-up for any other reason not listed above
	ED	Enrolment deviation

Annex C: Example of patient book

Patient ID: 3049

Treatment: AM + LUM
Date of Inclusion: 15 Sep 06
Gender: M
Age: 5,0 years
Weight: 15 kg
Last day with parasitaemia: 42
Antimalarial taken within 28 days before Inclusion: Unknown
Severe Malaria at Inclusion: Unknown

Gap is the number of missed days consecutive blood smear results

Table of Parasitaemia

Date	day	Pf	Pv	Po	Pm	Px	Temp	Gap
15 Sep 06	0	5 760	.a	.a	.a	.a	37,5 ° C	0 days
16 Sep 06	1	0	.a	.a	.a	.a	36,4 ° C	0 days
17 Sep 06	2	0	.a	.a	.a	.a	36,7 ° C	0 days
18 Sep 06	3	0	.a	.a	.a	.a	36,3 ° C	3 days
22 Sep 06	7	0	.a	.a	.a	.a	37,0 ° C	6 days
29 Sep 06	14	0	.a	.a	.a	.a	36,9 ° C	6 days
6 Oct 06	21	0	.a	.a	.a	.a	36,6 ° C	6 days
13 Oct 06	28	0	.a	.a	.a	.a	36,6 ° C	5 days
19 Oct 06	34	0	.a	.a	.a	.a	37,8 ° C	0 days
20 Oct 06	35	0	.a	.a	.a	.a	36,8 ° C	6 days
27 Oct 06	42	440	.a	.a	.a	.a	36,2 ° C	.l days

Table of Lab Results

Date	day	Hb	Htc	WBC
15 Sep 06	0	11,7	.a	.a
16 Sep 06	1	0,0	.a	.a
17 Sep 06	2	0,0	.a	.a
18 Sep 06	3	0,0	.a	.a
22 Sep 06	7	0,0	.a	.a
29 Sep 06	14	0,0	.a	.a
6 Oct 06	21	0,0	.a	.a
13 Oct 06	28	0,0	.a	.a
19 Oct 06	34	12,8	.a	.a
20 Oct 06	35	0,0	.a	.a
27 Oct 06	42	0,0	.a	.a

.a is for missing data where the variable(s) was not present in the source dataset
.b is for missing data in that particular patient record

Table of Unexpected Results

Day	Situation

Table of Deviations

Day	Deviation

Table of Efficacy related endpoints

Day	Endpoint
42	This is a LPF that occurred on D42
42	This is a LTF that occurred on D42
42	This is a Recrudescence
