# Supplementary material for: The Effect of Dosing Regimens on the Antimalarial Efficacy of Dihydroartemisinin-Piperaquine: A Pooled Analysis of Individual Patient Data
Source: PLoS Med. 2013 Dec 3;10(12):e1001564. doi: 10.1371/journal.pmed.1001564 (PMC3848996; doi:10.1371/journal.pmed.1001564)
Supplement: Text S5 — WWARN DP data and management statistical analytical plan. (PDF) [file pmed.1001564.s005.pdf]

Statistical Analysis Plan
DHA-PQP Dose Impact Study Group
Version 1.9

WorldWide Antimalarial Resistance Network (WWARN)

Suggested citation: Statistical Analysis Plan, DHA-PQP Dose Impact Study Group: Investigating the effect of mg/kg drug dosage on the clinical outcome

Version History

Version number	Revision(s) & reason for amendment	Release date
**V1.9**		**22.03.12**

WorldWide Antimalarial Resistance Network (WWARN)
www.wwarn.org

Contents

1. Introduction 3
2. Outline of Statistical Analysis 4
3. Statistical Methodology 7
4. Tools 9
5. References 10
6. Annex 11

1. Introduction

The efficacy of Artemisinin Combination Therapies (ACTs) is influenced by both the artemisinin derivative and the partner drug. Their role is to cure patients but also to prevent the survival and spread of artemisinin resistant strains of Plasmodium. Three of the most common partner drugs currently prescribed for the treatment of uncomplicated malaria are lumefantrine (LUM), piperaquine (PIP), and amodiaquine (AQ). The dosage of partner drugs must be sufficient to ensure that blood concentrations exceed the minimum inhibitory concentration of the parasite until all parasites are been killed. Although target doses are usually given as a total mg/kg over three days, in practice manufacturers' recommendations are often pragmatic and based upon weight "banding". This approach inevitably results in some patients at the margins of having either lower or higher dosages.

Young children are particularly vulnerable to extreme total dosages especially when drug administration is based on tablets (or fractions thereof) rather paediatric formulations or suspensions. The problem is further confounded when dosing is recommended according to age bands rather than actual body weight. Under-dosing has been suggested to play a role in the development of resistance (Terlouw *et. al.*, 2003). Preliminary modelling of dosing strategies according to known weight for age demographics in malaria patients, suggests a wide range of mg/kg dosing is used that may impact significantly on treatment efficacy and possibly safety.

Aim of the study

To determine the mg/kg dosing range of Dihydroartemesinin (DHA) and Piperaquine (PIP) adopted in clinical trials and investigate the effects of mg/kg dosing on clinical outcome.

Eligibility criteria for inclusion in pooled analysis

A study will be deemed eligible for the purpose of this analysis if they meet the following criteria:

- Prospective clinical efficacy studies of *P. falciparum* (either alone or mixed infections).
- Treatment with either **DHA-Piperaquine** with a minimum of 28 days of follow up.
- Data is available on exact dosage of piperaquine received by patients. Preferred but not essential information includes drug manufacturer, whether all doses were supervised, and whether drugs were administered with fat.
- PCR genotyping results classifying recrudescence failures from new infections.

The data sets uploaded to the WWARN repository will be standardized using the WWARN Data Management and Statistical Analysis Plans (DMSAP v1.1[1]) for clinical data and pooled into a single database of quality-assured individual patient data. Data will remain the property of the individual donor(s) and publication will be in accordance with an agreed publication plan (see publication policy document[2]).

2. Outline of Statistical Analysis

Baseline characteristics of the studies included in the analysis

An overall summary (overall study profile) of all the studies uploaded to WWARN repository will be presented and studies in which individual mg/kg dosing is available will be ascertained.

Baseline characteristics of studies eligible for the purpose of this project will be presented including information on transmission intensity, age, sex, weight, baseline parasitaemia, species (% of mixed infections) gametocytes on presentation, past history of malaria, haemoglobin, level of supervision of drug intake (full or partial) and on co-administration with fat. Haematocrit will be converted to haemoglobin using the following relationship:

$$Hematocrit\ (ht) = 5.62 + 2.60 * Haemoglobin\ (hb) \qquad \text{(Lee } et\ al.\text{, 2008)}$$

Assessment of heterogeneity and major differences between studies will be performed.

Study locations will be categorised into three strata according to known epidemiology: low, moderate and high transmission settings and also according to region (Africa, Asia, and S. America).
Transmission settings will be defined based on the triangulation of information given in the **publication**(s) (location, type of malaria transmission, use of bed nets, and month of study), **observed reinfection rate** and **expert opinion** through the discussion within the study group. Transmission intensity will be classified as low, moderate or high.

Diversity in dosing strategies

Descriptive statistics for different methods of dosing strategies (age based, weight based) will be calculated. Summary statistics of dosing strategies used in children (e.g. % studies using quarter/half tablets, % dissolving tablets, or paediatric formulation) will be reported.

The mg/kg distribution of artemisinin and PIP will be presented using box plots or histograms together with their descriptive statistics.

Scatter plot of mg/kg dosing (Y axis) and age (X axis).

Scatter plot of mg/kg dosing (Y axis) and weight (X axis).

Treatment adherence will be reported as a binary variable (1= Yes, 0=No) as well by degree (eg the number of drop outs and if possible missed doses). A patient will be classified as treatment adherent if all the full course treatment has been taken.

[1] http://www.wwarn.org/partnerships/data/methodology/clinical
[2] http://www.wwarn.org/sites/default/files/PublicationPolicy.pdf

Efficacy endpoints

Primary: PCR adjusted risk of *P. falciparum* recrudesence

Secondary: PCR adjusted risk of *P. falciparum* reinfection
PCR unadjusted risk of *P. falciparum* recurrence
Risk of *P. vivax* recurrence
Parasite clearance

Tertiary: Risk of gametocyte carriage during follow up
Haemoglobin measurements over time

All the primary and secondary endpoints on interest will go through the same analysis as outlined below. Analysis of tertiary endpoint will be carried out provided enough data is present; else, only summary statistics will be reported.

For the cumulative risk of recurrence (*P. falciparum* adjusted, *P. falciparum* unadjusted, and *P. vivax*), survival and Cox regression analysis will be used (see 3.2 and 3.3 below). Definitions of status and censorship are detailed on page 14 of the Clinical Module DMSAP v1.1. In addition, the median time to presentation with recurrent infection will be calculated.

For the parasite clearance, the proportions of patients cleared at day 1, 2 and 3 will be assessed. Definitions are detailed on page 15 of the Clinical Module DMSAP v1.1. The proportions of patients with positive parasitaemia at day 1, 2 and 3 (Parasite Positivity Rates, **PPRs**) will be calculated for each individual studies with confidence intervals derived using Wilson's method. Fleiss's method will be used to construct 95% confidence intervals associated with the overall pooled estimate, where the aggregate data (for the stratification of interest) came from more than one study. The effect of PIP and DHA dosage on the risk of parasite positivity will be assessed using generalised linear mixed model with study sites fitted as a random effect and by specifying logit link function.

Gametocyte carriage will be assessed as the proportion of patients with P. falciparum gametocytes on day 3, 7, 14 or 21, (GPR - Gametocyte Positivity Rates). Fleiss's method will be used to construct the 95% confidence intervals for GPR. Summary outcome measures will be stratified by whether or not gametocytes were present on admission. Logistic regression with logit link will be used to assess the univariate and multivariate risk factors for gametocytes carriage during the follow-up. Similar model selection strategy will be employed as in Section 3.3 later for building multivariate model.

Haemoglobin measures (continuous) will be analysed using linear model with study effects fitted as random. It would be adjusted for time and other baseline covariates.

ROC analysis (or logrank statistics) will be used to define a breakpoint for mg/kg with greatest discrimination. This will be carried out by categorising each patient as Failure (1/0) on day 28 (or day 42). Patients who were lost to follow up will be excluded. The mg/kg cut offs will be defined separately for PCR adjusted *P. falciparum*, unadjusted *P. falciparum*, *P. falciparum* reinfection, and *P. vivax* recurrence.

Risk factors for recurrent parasitaemia (PCR adjusted recrudescence and new infection)
Univariate analysis of confounding factors (adjusted for the study effects) associated with the primary and secondary endpoint of interests (Section 2.3) will be conducted. The available confounders of interest are age, sex, baseline parasitaemia, baseline anaemia, mixed infections, vomiting of drug within 1 hour, and transmission zones (Low, Moderate, and High) site. The latter will include transmission intensity as a proxy of global immunity and age as a marker of host immunity. For risk factors for PCR adjusted recrudescence, known confounders (age and parasite density at enrolment) will be forced into the model regardless of statistical significance. For the risk of PCR adjusted new infection, age and transmission intensity will be forced into the model along with any covariates that were significant at a 10% level of significance in the univariate analysis.
Cox regression analysis of mg/kg dose partner drug (as defined in 2.3.4) as a risk factor for the four recurrence outcomes, after controlling for known confounding factors identified in 2.4.1 will be carried out. Median (IQR) mg/kg dose of artemisinin derivatives or partner drugs in patients failing treatment (i.e. recurrence) vs successfully treated (defined as reaching the end of the study duration without failure) will be calculated.

Population Attributable Risk (PAR) of low mg/kg dose on risk of recurrence i.e. what proportion of recurrences were attributable to poor dosing will be investigated. The PAR% will be computed using formula first proposed by Levin [Levin 1953] based on the prevalence of the risk factor in population (p_i) and the relative risk (RR) estimate for the risk factor.

$$PAR_{Levin} = \frac{p_i(RR-1)}{1+p_i(RR-1)} * 100$$

The relative risks (RR) will be replaced by the adjusted hazard ratio (AHR) from the multivariable cox's regression model [Benichou, 2001]. Continuous risk factors will be categorised to compute the PAR associated with exposure to those risk factors with baseline parasitaemia categorised at >= 100,000/µL. An overall PAR will be calculated as 1 minus the product of 1-each of the individual PAR [Price et al. 2001] assuming the risk factors are independent.

$$Overall\ PAR = 1 - \prod_{r=1}^{R}(1-PAR_r)$$

Time to recrudescence (median) will be calculated for different age-groups for the patients who failed. The proportion of patients who failed before day28 or between day28 and day42 will be presented.

The effect of the level of supervision on the risk of recurrence will be assessed. The efficacy outcomes for the partially, fully and not supervised groups will be computed.

Analysis of risks factors for being underdosed and public health importance

Investigation will be carried out to assess the risk factors for a patient receiving a low mg/kg dose (as defined in 2.3.4). The effect of age, sex, protocol type, target dose, adherence violations, location and any other available risk factors will be assessed.

Global implications

The WHO weight-for-age reference population database (**if available**) will be used to calculate the weight for the patients with missing body weights.

The mg/kg of drug dosing will be plotted on the Y-axis and age will be plotted on the X-axis. Proportion of underdosed patients $(\widehat{P_u})$ i.e. the patients who received less than the recommended therapeutic ranges for anti-malarial will be estimated.

The estimated proportion of underdosed patients can then used to estimate the number of patients who failed treatment due to the under dosing globally.

3. Statistical Methodology

Descriptive statistics

Normality of the distribution of the drug dosages (mg/kg total) will be checked using a Q-Q plot. The p-value from Shapiro-Wilk's normality test will be reported.

The mean, 10th, 25th, 75th, 90th percentile and the median mg/kg dose will be calculated for each dosing group (under/below as defined in Section 2.3.4). The distribution of the drug dosages will be presented graphically (histogram, box plots).

Patients will be categorised into four age groups ≤1 year, >1-≤5 years, >5-≤12 years and >12 years. The summary statistics will be further broken down by gender and age category.

Survival analysis

PCR Adjusted and Unadjusted outcomes (ref: DMSAP v1.1 for definitions) obtained using WWARN standardised outputs will be used to compute the Kaplan-Meier (K-M) estimates for the two dosing groups. The K-M estimates will be presented graphically together with the associated tables. Log rank test stratified by study will be performed at 5% level of significance to test if the K-M profiles are significantly different from each other.

Frailty analysis will be carried out (specifying study effect as random) to adjust for the differences between the study sites (Glidden and Vittinghoff, 2004). Univariate and multivariate analysis of risk factors associated with the primary and secondary endpoint (PCR adjusted new infection) of interest will be conducted using Cox's proportional hazards regression model with shared frailty on study sites to account for unobserved heterogeneity.

Model selection for determinants

Model building will be carried out first by investigating if any of the available variables (Annex A.1) are related to the treatment outcome using Cox's regression model. Any known confounding factors will be forced into the model even if they are statistically non-significant. All the variables which were significant in the univariate analysis at 10% level of significance will be kept for the multivariate analysis.

Model with known confounders will be fitted first (baseline model). Variables and covariates will then be added to the baseline model and the Likelihood Ratio Test (LRT) i.e. changes in log likelihood $(-2\ Log\hat{L})$ will be compared (for nested models) to identify the variables which results in a significant reduction in $-2\ Log\hat{L}$ at 10% level of significance. Akaike's Information Criterion (AIC) will be used to compare competing non-nested models; models with smaller AIC will be preferred.

Inclusion of covariates in the final model will be based on their effect on model coefficients and the degree to which they improved the overall model (based on a likelihood ratio test).The final model will then be used to estimate the hazards ratio (HR) for under-dosed group (optimal cutoff derived from the data) relative to the normally dosed group of patients. Cox-Snell's residuals , Martingale's residuals and Schoenfeld's residuals will be examined to determine the appropriateness of model fit.

Proportional hazards (PH)

Schoenfeld residuals against transformed time for the dosing group will be used to determine if the assumptions of PH across different dosing groups are reasonable. Any systematic departures from horizontal lines are indicative of non-proportional hazards (Schoenfeld, 1982). In addition to the Schoenfeld residuals plot, a global and individual chi-squared test will be used to test the assumption of proportional hazards. Any violation of the assumption of proportionality was reported.

If the K-M profiles for different groups crosses over then the assumption of PH won't be tested. Any further covariates will be categorised in order to test the assumptions of PH.

Finding the optimal cut points

The value of the mg/kg drug dosage which maximizes the log-likelihood statistics in Cox's model will be identified as the optimal threshold based on 1,000 bootstrap samples. This will be carried out separately at day28, day42 and day63.

The use of Logrank statistics to define the breakpoint will be explored. The value of the drug dosage which maximizes the logrank statistics will be identified as the optimal threshold (Contal and O' Quigley, 1999). The suitability of Receiving Operating Characteristics Curves (ROCs) for finding the optimal cutpoints for mkg/kg drug dosage which best differentiates the PCR adjusted failure will be explored. The optimal cutoff point will be defined as the maximum value of Youden's Index (*J*), which is sensitivity+ specificity-1 (Price *et al.*, 2007). Alternative methods for determining the optimal cutoff will also be explored (e.g. data driven methods such as percentiles). Outcome oriented approaches will also be explored.

Predicting dose to achieve 95% efficacay

In final multivariate model, the predicted effect of increasing the drug dose of piperaquine (mg/kg) will be calculated for every 5 unit increase starting from 30 mg/kg. The 95th percentile of predicted risk will then be computed for each mg/kg dosage of PIP with all the variables keeping their values for the covariates evaluated at average random effect. The dose which results in the 95th percentile of the predicted risk to be <5% i.e. estimated efficacy to be >=95% will be reported. The suitability of Emax models to define the dose to achieve 95% clincail efficacy will be explored.

4. Tools

All statistical analyses will be carried out using R 2.14.0 released on 2011-10-31 by The R Foundation for Statistical Computing. However, when equivalent statistical methods are applied, changing the use of statistical software does not require amendment of this SAP.

5. References

Collett, D. (2003) Modelling Survival data in Medical Research. Second edition. Boca Raton: Chapman and Hall/CRC.

Contal, C., O'Quigley, J. "An application of changepoint methods in studying the effect of age on survival in breast cancer," Computational Statistics and Data Analysis, 1999; 30, 253 - 270.
Stable URL: http://www.sciencedirect.com/science/article/pii/S0167947398000966
Accessed: 15/01/2012

Glidden, D.V., and Vittinghoff, E. "Modelling clustered survival data from multicentre clinical trials". Statistics In Medicine.Statist. Med. 2004; 23:369–388 (DOI: 10.1002/sim.1599)
Stable URL: http://onlinelibrary.wiley.com/doi/10.1002/sim.1599/pdf
Accessed: 27/03/2012

Mandrekar JN , Mandrekar SJ , Cha SS . Cutpoint determination methods in survival analysis using SAS®. Proceedings of the 28th SAS Users Group International Conference (SUGI), 2003; 261-28.
Stable URL: http://www2.sas.com/proceedings/sugi28/261-28.pdf
Accessed: 15/01/2012

Lee, S.J, Stepniewska ,K., Anstey, N., Ashley, E., Barnes, K., Binh, T.Q., D'Alessandro, A., Day, N.P.J., de Vries, PJ., Dorsey,G., Guthmann, JP., Mayxa, M., Newton,P., Nosten, F., Olliaro,P., Osario,L., Pinoges,L., Price,R., Rowland,M., Smithuis,F., Taylor,R., White,N.J. (2008). "The relationship between the haemoglobin concentration and the haematocrit in Plasmodium falciparum malaria". Malaria Journal 2008, 7:149 doi: 10.1186/1475-2875-7-149.
Stable URL: http://www.malariajournal.com/content/7/1/149
Accessed: 21/03/2012

Price, R.N., Hasugian,A.R., Ratcliff,A., Siswantoro,H., Purba, H.L.E., Kenangealem,E., Lindegardh,N., Penttinen,P., Laihad,F, Ebsworth,E.P., Anstey,N.M., and Tjitra, E. (2007). "Clinical and Pharmacological Determinants of the Therapeutic Response to Dihydroartemisinin-Piperaquine for Drug-Resistant Malaria". *Antimocrobial Agents and Chemotherapy*, Nov.2007, Vol.51, No.11, p.4090-4097.
Stable URL: http://aac.asm.org/content/51/11/4090.full.pdf
Accessed: 21/03/2012

Schoenfeld ,D (1982). "Partial residuals for the proportional hazards regression model". Biometrika, Vol. 69, No. 1. (Apr., 1982), pp. 239-241.
Stable URL: http://www.jstor.org/stable/2335876
Accessed: 14/12/2011 12:40

Terlouw, D.J., Nahlen, B.L.,Courval,J.M., Kariuki,S.K., Rosenberg,O.S., Oloo,A.J., Kolczak,M.S., Hawley,W.A., Lal,A.A., Kuile, F.O. (2003). "Sulfadoxine-Pyrimethamine in Treatment of Malaria in Western Kenya: Increasing Resistance and Underdosing". Antimicrobial agents and chemotherapy, sept. 2003, p. 2929–2932
Stable URL: http://aac.asm.org/content/47/9/2929.short
Accessed: 21/01/2012 12:40

Levin ML (1953) The occurrence of lung cancer in man. Acta Unio Int Contra Cancrum 9: 531-541.

6. Annex

A.1 List of available covariates

Description	Type
WWARN Status for Pf Adj	Primary Response
WWARN Status for Pf UnAdj	Primary Response
ETF	Secondary Response
LCF	Secondary Response
LPF	Secondary Response
LTF	Secondary Response
Early LTF (before D14, no PCR)	Secondary Response
History of Fever (0/1) at inclusion	Baseline Variable
Severe Malaria at inclusion	Baseline Variable
Haemoglobin at inclusion	Baseline Variable
Falciparum density at Inclusion	Baseline Variable
Gamf (/µL) at inclusion	Baseline Variable
Max Temp Day0	Baseline Variable
D0 Ht<20%	Baseline Variable
Age in Years	Available Variable
Gender	Available Variable
Weight	Available Variable
Antimalarial in last 28 days	Available Variable
Parasite density at Inclusion	Available Variable
Max Falciparum Asexual parasitaemia on Day1	Available Variable
Max Falciparum Asexual parasitaemia on Day2	Available Variable
Max Falciparum Asexual parasitaemia on Day3	Available Variable
Max Temp Day1	Available Variable
Max Temp Day2	Available Variable
Max Temp Day3	Available Variable
Dosing method (single day, broken down over days etc.)	Available Variable
Total mg/kg dose at each day of dosing regimen	Available Variable
Total mg/kg dose during course	Available Variable
